# Amplification-Free Electrochemiluminescent Biosensor for Ultrasensitive Detection of *Fusobacterium nucleatum* Using Tetrahedral DNA-Based CRISPR/Cas12a

**DOI:** 10.34133/cbsystems.0266

**Published:** 2025-05-01

**Authors:** Xindan Zhang, Minkang Wu, Haoran Shi, Soochan Kim, Shixiang Lu, Ping Wang, Jieling Qin

**Affiliations:** ^1^ School of Chemistry and Chemical Engineering, Beijing Institute of Technology Zhengzhou Academy of Intelligent Technology, Beijing Institute of Technology, Beijing 100081, China.; ^2^ Tongji University Cancer Center, Shanghai Tenth People’s Hospital, School of Medicine, Tongji University, Shanghai 200092, China.; ^3^ School of Bioengineering, Dalian University of Technology, Dalian City, Liaoning 116024, China.; ^4^ School of Chemical Engineering, Sungkyunkwan University, Seoul, South Korea.

## Abstract

*Fusobacterium nucleatum*, a bacterium linked to colorectal cancer, possesses a specific gene called *fadA* that serves as an early diagnostic biomarker. The CRISPR/Cas12a system has demonstrated marked potential for nucleic acid detection due to its satisfactory selectivity and trans-cleavage ability. However, most CRISPR/Cas-based sensors suffer from problems such as probe entanglement or local aggregation, reducing the Cas enzyme efficiency. In this study, an amplification-free biosensing platform for ultrasensitive detection of *F. nucleatum* was developed by integrating the highly specific CRISPR/AsCas12a with an improved electrochemiluminescence (ECL) biosensor. Different from the conventional 1- or 2-dimensional probes, the platform was constructed by tetrahedral DNA nanostructure (TDN) probes conjugated with quenchers and coralliform gold (CFAu) functionalized with luminescent agents. The TDN serves as an exceptional scaffold to modulate the recognition unit, substantially enhancing the recognition and cleavage efficiency of AsCas12a toward the probes. Furthermore, the high surface area of CFAu provides extensive landing sites for the luminescent agents, thereby improving the detection sensitivity. The prepared ECL biosensor exhibited a wider linear range (10 fM to 100 nM) and was capable of detecting *F. nucleatum* down to 1 colony-forming unit/ml. Additionally, the high mismatch sensitivity of AsCas12a to protospacer adjacent motifs and nearby areas provides a strategy for distinguishing mutant from wild-type sequences. Finally, by designing CRISPR RNA (crRNA), this diagnostic method can also be easily modified to detect other bacteria or biomarkers for the early diagnosis of various diseases.

## Introduction

Colorectal cancer (CRC) is currently one of the most prevalent malignancies [1]. Compared to healthy individuals, CRC patients exhibit reduced bacterial diversity in their intestinal mucosa and fecal samples, with notable changes in certain bacterial populations [2,3]. Among them, *Fusobacterium nucleatum* plays a role in various stages of CRC progression and accelerates tumorigenesis [4,5]. *F. nucleatum*, a clostridial anaerobic Gram-negative bacterium found in the gut and oral flora, can enter the bloodstream through periodontal infections, migrate to the gut, and participate in the development of CRC by hematogenous or digestive tract swallowing. *F. nucleatum* can secrete multiple virulence factors, including Fusobacterium adhesin A (FadA), encoded by the gene *fadA*, which is found at elevated levels in CRC patients’ fecal macrogenome [6,7], with a high affinity for cadherin on the surface of CRC cells, enhancing the attachment of *F. nucleatum* to CRC cells and avoiding clearance by the immune system [8]. Therefore, quantitative detection of *F. nucleatum*-specific gene *fadA* in fecal samples is a promising approach for early CRC diagnosis, which is essential to improve patient survival [9].

The clustered regularly interspaced short palindromic repeats (CRISPR)-based diagnostic methods have become popular strategies for pathogen nucleic acid detection because of their high sensitivity, specificity, and reliability [10]. Various CRISPR/Cas-based methods, especially CRISPR/Cas9, CRISPR/Cas12, and CRISPR/Cas13, have been developed for DNA detection [11]. Among them, Cas12a is notable for its ability to detect DNA without reverse transcription. Cas12a can be activated with trans-cleavage activity after recognizing double-stranded DNA (dsDNA) to indiscriminately cleave single-stranded DNA (ssDNA) that serves as a reporter molecule, which can increase sensitivity and thus allow for robust signal amplification [12–14]. Current bacterial detection methods using the CRISPR/Cas system include fluorescence, electrochemical biosensors, and lateral flow assays (LFAs) [11]. Specifically, fluorescence methods have attracted wide attention for their simplicity, high efficiency, easy signal amplification (in combination with polymerase chain reaction (PCR) or isothermal amplification techniques), and visualization of readings, but require costly fluorescence readers and lengthy processes [15]. LFAs are portable and inexpensive but lack efficiency and quantitative capability [16,17]. Electrochemical biosensors offer specificity, affordability, miniaturization, portability, and rapid results, showing great promise for innovative bacterial detection methods [18]. For instance, Li et al. [18] developed a highly sensitive electrochemical sensor using CRISPR/Cas12a and isothermal amplification for detecting Listeria monocytogenes through the nonspecific cleavage of the probe by activated Cas12a. Utilizing hairpin DNA-MB modified on the electrode, Chen et al. [19] developed a CRISPR/Cas12a and rolling cycle amplification (RCA)-based electrochemical platform for sensitive detection of pathogenic bacteria. Liu et al. [20] employed a hybridization chain reaction (HCR)-based CRISPR-Cas12a on a similar sensor to detect *Salmonella typhimurium* effectively. However, probes on these electrodes can suffer from issues like entanglement or localized aggregation to reduce the Cas enzyme efficiency and sensing performance [21].

**Fig. 1.**
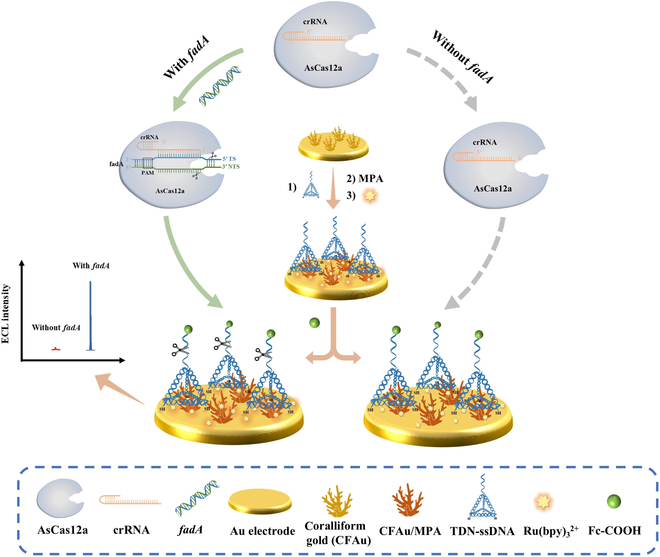
Schematic diagram of an amplification-free electrochemical biosensor for detecting *F. nucleatum* (*fadA*) based on the CRISPR/Cas system and tetrahedral DNA nanostructures (TDNs). Coralliform gold (CFAu) nanostructures are modified on the electrode surface by electrochemical deposition followed by immobilizing TDN-ssDNA through sulfur–gold bonding. 3-Mertopropionic acid (MPA) self-assembled monolayer (SAM) is then formed on the electrode surface and the luminescent reagent ruthenium tris(bipyridine) [Ru(bpy)_3_^2+^] is soft-landed. In addition, ferrocene carboxylic acid (Fc-COOH) is immobilized at the TDN-ssDNA end by amide reaction for quenching Ru(bpy)_3_^2+^. In the presence of *fadA*, the trans-cleavage activity of AsCas12a is activated and cleaved fluorescent and tetrahedral probes, resulting in intense electrochemiluminescence (ECL). However, in the absence of *fadA*, the ECL intensity is very weak. Thus, the specific detection of *fadA* and *F. nucleatum* is achieved.

In this study, we developed an amplification-free electrochemiluminescence (ECL) biosensor using the CRISPR/Cas system for ultrasensitive detection of the specific gene *fadA* from *F. nucleatum* at femtomolar levels (Fig. 1 and Fig. S1). ECL has emerged as a sensitive method for detecting small molecules, nucleic acids, and proteins [22–24], due to its superior spatiotemporal control and virtually undetectable background noise [25,26]. In ECL, luminescent reactants on the electrodes are regenerated through electrochemical reactions, allowing them to repeatedly participate in ECL reactions with remaining co-reactants. This ongoing photon production during each measurement cycle substantially enhances the sensitivity of ECL. Specifically, the purified AsCas12a, combined with the CRISPR RNA (crRNA), designed for navigation, forms the CRISPR/Cas system with high trans-cleave efficiency and specific recognition function. Additionally, tetrahedral DNA nanostructures (TDNs) with tip-modified ssDNA (TDN-ssDNA) were also harnessed for their rigidity, stability, and thiol groups at the bottom that strongly anchor to gold electrode surfaces through sulfur–gold bonds (S–Au). This allows the recognition sequences to have appropriate spatial distribution and molecular orientation, enhancing Cas12a recognition and increasing sensing performance [21,27,28]. For electrode modification, the coralliform gold (CFAu) nanostructures were electrochemically synthesized on the electrode followed by the immobilization of TDN-ssDNA via Au–S, and then self-assembled 3-mercaptopropionic acid (MPA) on the surface, which aids in the soft landing of the luminescent reagent ruthenium tris(bipyridine) [Ru(bpy)_3_^2+^]. The large surface area of CFAu provides more possibilities for the modification of TDN-ssDNA and Ru(bpy)_3_^2+^ on the electrode surface. In addition, the luminescence quencher ferrocene carboxylic acid (Fc-COOH) was immobilized at the end of TDN-ssDNA to quench Ru(bpy)_3_^2+^. Luminescence was also achieved by nonspecific cleavage of the single-stranded probes on TDN-ssDNA after activation of AsCas12a enzyme by *fadA*, thus enabling specific detection of *fadA* and *F. nucleatum* (Fig. 1). Moreover, we compared this method [detection range of 10 fM to 100 nM for *fadA* and 1 to 10^8^ colony-forming units (CFU)/ml for *F. nucleatum*] with fluorescence (detection range of 0.078 to 2.5 nM for *fadA* and 39,063 to 1,250,000 CFU/ml for *F. nucleatum*), further demonstrating its excellent analytical performance. We believe that this ultrasensitive gene-based detection platform holds considerable promise in CRC diagnosis.

## Materials and Methods

### Construction, expression, and purification of AsCas12a

pX330-AsCas12a was kindly provided by D. Li. Then, the prokaryotic expression vector pET28a-AsCas12a with His tag was constructed through homologous recombination. The plasmids were transformed into BL21 competent cells, and isopropyl-β-d-thiogalactopyranoside (IPTG) was added at an OD600 (optical density at 600 nm) value of 0.6 to 0.8 to induce protein expression. After bacterial lysis, the supernatant was collected and incubated with Ni-NTA beads from Changzhou Smart-Lifesciences Biotechnology Co. Ltd., (Jiangsu, China) to collect the protein. Protein expression and collection were then confirmed using sodium dodecyl sulfate–polyacrylamide gel electrophoresis (SDS-PAGE). Further purification of AsCas12a was achieved through molecular sieving, and the purified protein was stored at −80 °C after ultrafiltration concentration.

### Design and synthesis of crRNA

The function of the CRISPR/Cas12a system requires crRNA to guide the Cas12a enzyme to recognize the protospacer adjacent motifs (PAMs), which allows the enzyme to bind to dsDNA molecules and cleave them. The PAM sequence of AsCas12a enzyme was identified as 5′-TTTN-3′ (N = any nucleotide) [29]. The crRNA was designed based on the protospacer sequence following the PAM sequence. This involved identifying the contiguous TTTN sequence on *fadA* and selecting the subsequent 20 nucleotides (nt). In this study, Benchling website (https://www.benchling.com/) and CHOPCHOP website (https://chopchop.cbu.uib.no/) were utilized to design the crRNA. Upon entering the target gene and selecting the crRNA type, dozens of different crRNA sequences were generated. After evaluating their off-target scores, guanine and cytosine (GC) content, self-complementarity, efficiency, and number of mismatches, 5 sequences were selected for this study. Their corresponding protospacer sequences were then analyzed using Basic Local Alignment Search Tool (BLAST) on National Center for Biotechnology Information (NCBI), selecting the sequence with optimal specificity (5′-UUCUGGAGCUGCUAACAAUU-3′). After that, the selected sequence was combined with the corresponding hairpin structure sequence (5′-UAAUUUCUACUCUUGUAGAU-3′) to create the full crRNA sequence (5′-UAA​UUUCUACUCUUGUAGAUUUCUGGAGCUGCU​AACAAUU-3′).

### TDN formation

Four single-stranded sequences (S1, S2, S3, S4-ssDNA) of TDN were designed on the NUPACK website (https://nupack.org/), synthesized by Sangon Biotech (Shanghai) Co. Ltd. and stored at −20 °C. The formation of TDNs was referred to in the literature [30] and then confirmed by 3% agarose gel electrophoresis at 100 V for 60 min. Specifically, S1, S2, S3, and S4-ssDNAs were mixed in a buffer (10 mM tris–HCl, 5 mM MgCl_2_, pH 8.0) to a final concentration of 1 μM each, with tris (2-carboxyethyl) phosphine (TCEP) added to a final concentration of 500 μM, and gently vortexed. The 5′ terminus of the 64-nt ssDNA is equipped with 6 carbon-linked sulfhydryl groups (-SH), while the 5′ terminus of the 84-nt ssDNA carries 6 carbon-linked amino groups (-NH_2_). The solution was denatured by heating at 95 °C for 10 min, then rapidly cooled to 4 °C, and maintained at 4 °C for 30 min to preserve the TDN structure. This complementary pairing of the 4 bases engenders a tetrahedral structure (TDN-ssDNA) that exhibits mechanical rigidity and structural stability. One vertex of this tetrahedron features an overhanging ssDNA probe with an -NH_2_ modification, which is subsequently utilized to attach a quenching agent. The remaining 3 vertices have -SH groups, which can be covalently bound to the gold electrode surface through S–Au interactions.

## Results and Discussion

### Characterization and verification of CRISPR/Cas12a system

The pET28a vector and AsCas12a fragment were PCR-amplified (Fig. S2A), and the prokaryotic expression plasmid pET28a-AsCas12a was then constructed via homologous recombination in Fig. S2B. After transforming the plasmid into BL21 competent cells, the optimal conditions for protein induction were established at 0.1 mM IPTG and 25 °C (Fig. S2C). The His-tagged AsCas12a protein was bound to Ni-NTA beads and eluted using a high concentration of imidazole. As shown in Fig. S3A, the eluted sample was verified by gel electrophoresis, showing the successful elution of a substantial amount of protein with an approximate molecular weight of 152 kDa. Following dialysis and ultrafiltration, the AsCas12a protein was further purified using a molecular sieve and confirmed by SDS-PAGE (Fig. S3B), in which the second peak, identified as the target protein, was collected, yielding AsCas12a with high purity.

**Fig. 2.**
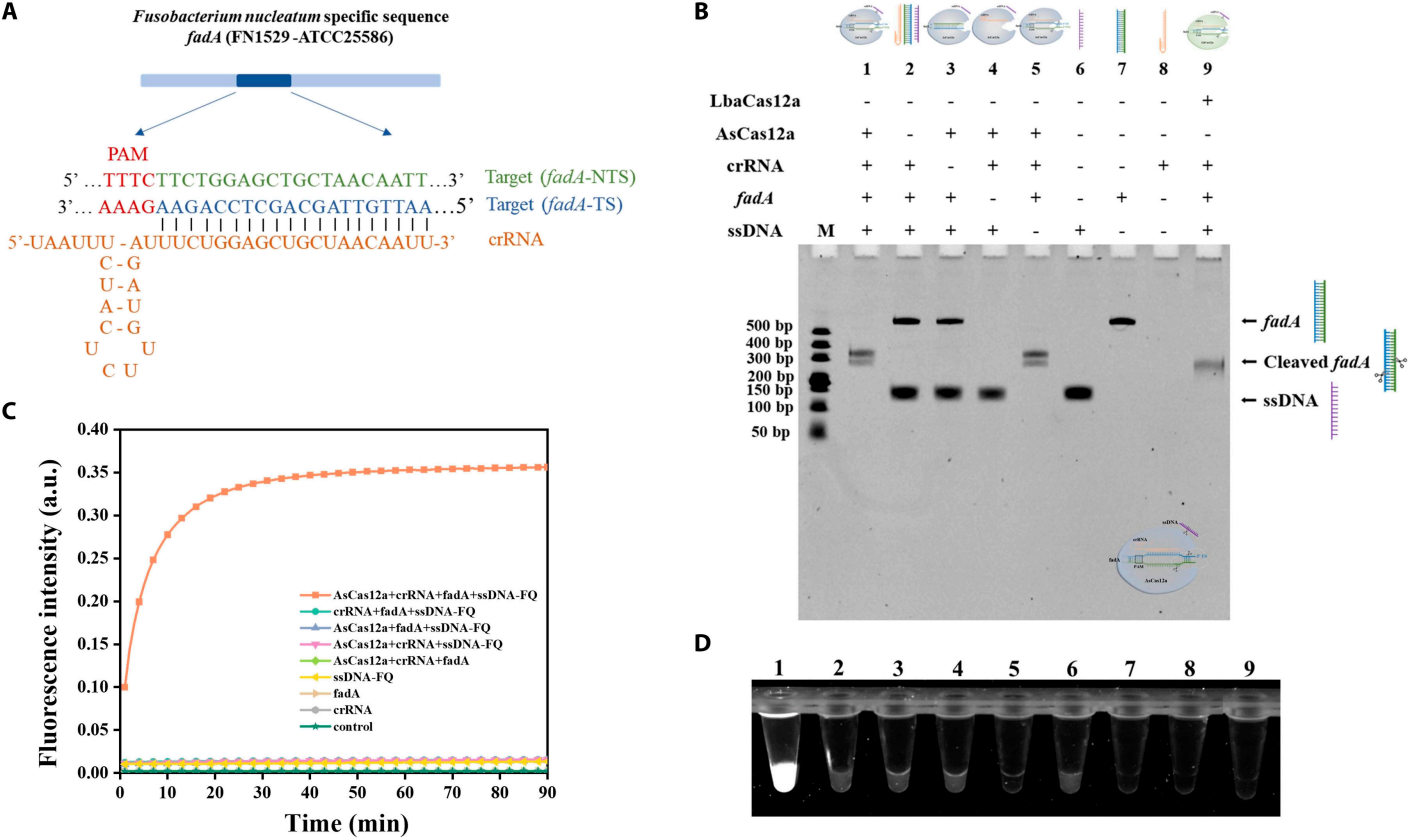
Characterization and verification of CRISPR/Cas12a system. (A) Design of crRNA sequence and binding to the *F. nucleatum*-specific *fadA* sequence. PAM, protospacer adjacent motifs; TS, target strand; NTS, nontarget strand. (B) The 8% urea denaturing PAGE analysis of AsCas12a’s cis-cleavage and trans-cleavage activities. (C) Real-time fluorescence analysis of the feasibility of the CRISPR/Cas12a system for nonspecific cleavage of ssDNA. (D) Schematic representation of the system under blue light after 90 min of reaction using the fluorescence method. (1) AsCas12a + crRNA + *fadA* + ssDNA-FQ; (2) crRNA + *fadA* + ssDNA-FQ; (3) AsCas12a + *fadA* + ssDNA-FQ; (4) AsCas12a + crRNA + ssDNA-FQ; (5) AsCas12a + crRNA + *fadA*; (6) ssDNA-FQ; (7) *fadA*; (8) crRNA; (9) control.

The crRNA was then designed for the recognition of the *fadA* gene, a dsDNA target for the detection of *F. nucleatum* due to its specificity. Briefly, the protospacer sequence was identified by searching for the fragment 5′-TTTN-3′ in the *fadA* gene. The corresponding hairpin structure sequence was then used to construct the complete crRNA (Fig. 2A). For the verification of the CRISPR/Cas12a system, the AsCas12a/crRNA structure was formed by mixing AsCas12a and crRNA in NEBuffer 2.1 followed by incubation at room temperature for 15 min. An 8% urea denaturing PAGE was employed for the investigation of the enzymatic activity, with LbaCas12a serving as a control. As shown in Fig. 2B, the cleaved *fadA* fragment and the complete disappearance of ssDNA sequence were only observed when the AsCas12a/crRNA/*fadA* complex was formed (lane 1) compared to the group lacking AsCas12a (lane 2), crRNA (lane 3), or *fadA* (lane 4), indicating that *fadA* can be effectively recognized by AsCas12a/crRNA upon enzymatic scanning of the PAM sequence and the guidance of crRNA with a 20-nucleotide spacer, which led to the successful initiation of trans-cleavage activity of AsCas12a, cleaving nontarget ssDNA reporters in lane 1. Moreover, the result of LbaCas12a group (lane 9) similar to AsCas12a further confirmed that the presence of the *fadA* gene can activate the trans-cleavage activity, and cut nontarget ssDNA reporters, indicating a potential enhancement in the specificity and sensitivity of electrochemical detection based on the AsCas12a/crRNA system.

The possible mechanism by which the Cas12a/crRNA complex identifies and cleaves the target strand (TS) has been previously documented [12,31]. In this study, the AsCas12a enzyme initially scans the *fadA* gene in a one-dimensional hopping manner. Upon encountering a potential target site containing a PAM sequence (5′-TTTN-3′), the crRNA within the complex pairs with the TS through base complementarity, initiating R-loop formation. If there is a sufficient match (>17 base pairs), a stable R-loop forms, activating the RuvC domain of AsCas12a for DNA cleavage. This activation leads to the generation of interleaved dsDNA breaks at the 5′ and 3′ ends of the distal PAM. Subsequently, AsCas12a releases the *fadA* cleavage product at the distal PAM end while remaining bound to the dsDNA cleavage product proximal to the PAM in a catalytically active, trans-active state. Notably, the nontarget strand (NTS) of the dsDNA, as shown in (Fig. 2A), is crucial in stabilizing the AsCas12a/crRNA complex, ensuring that it maintains the optimal conformation for trans-cleavage of ssDNA.

Furthermore, the ssDNA-FQ (FAM-TTAATT-BHQ1) was utilized for real-time fluorescence monitoring of AsCas12a’s trans-cleavage activity. As illustrated in Fig. 2C and D, only the AsCas12a/crRNA/*fadA* group displayed a marked increase in fluorescence intensity compared to the group without AsCas12a, crRNA, or *fadA*, indicating the essential role of the AsCas12a enzyme, crRNA, and target sequence in the functionality of the CRISPR/Cas system.

### Preparation and characterization of TDN-ssDNA and CFAu

**Fig. 3.**
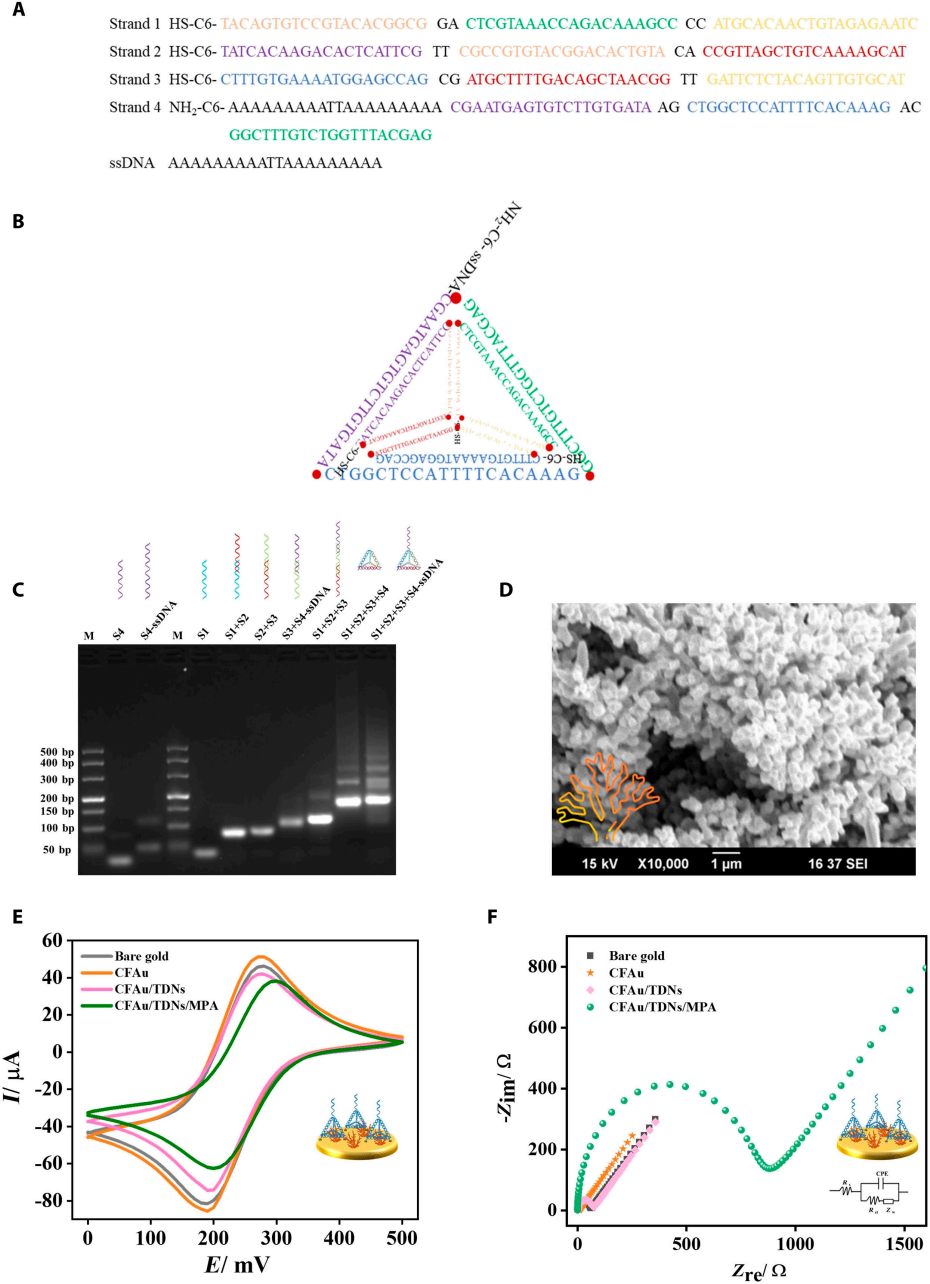
Preparation and characterization of TDN-ssDNA and CFAu. (A) Sequences of three 64-nt DNA strands with sulfhydryl modification at the 5′ end and one 84-nt DNA strand with amino modification at the 5′ end. (B) Sulfhydryl-modified TDNs with 20-nt single-stranded DNA (ssDNA) at the tip formed by base complementary pairing of the 4 single strands of DNA in (A). (C) The 3% agarose gel electrophoresis analysis of single stands, partial assembly combinations, TDN (S1 + S2 + S3 + S4), and TDN-ssDNA (S1 + S2 + S3 + S4-ssDNA). (D) Scanning electron microscopy (SEM) image of CFAu. (E) Cyclic voltammetry (CV) measurements for bare gold, CFAu-modified, CFAu/TDN-modified, and CFAu/TDN/MPA-modified electrodes with applied potential of 0 to 0.5 V in 20 mM Fe (CN)_6_^3−/4−^/PBS (pH 7.4) at a scan rate of 10 mV s^−1^. (F) Electrochemical impedance spectroscopy (EIS) measurements for bare gold, CFAu-modified, CFAu/TDN-modified, and CFAu/TDN/MPA-modified electrodes with the frequency range of 0.1 Hz to 100 kHz in 20 mM Fe (CN)_6_^3−/4−^/PBS (pH 7.4). *R*_S_, *R*_et_, CPE, and *Z*_w_ denote the solution resistance, electron transfer resistance, double-layer capacitance, and Warburg diffusion resistance, respectively.

The construction of TDN-ssDNA involves the base complementary pairing of three 64-nt ssDNA fragments and one 84-nt ssDNA fragment, each at equimolar concentrations (Fig. 3A and B) [30]. This strategic anchoring of TDN-ssDNA not only prevents probe detachment but also considerably enhances mass transport efficiency. Compared to 1- or 2-dimensional probes, TDN-ssDNA serves as an exceptional scaffold for modulating the recognition unit, allowing more precise control over the density and orientation of the reporter probes. Furthermore, TDN-ssDNA minimizes nonspecific adsorption on the electrode surface and facilitates a solution-like environment [27]. Thus, TDN-ssDNA markedly increases the likelihood of interactions and reactions between AsCas12a/crRNA and ssDNA probes. Fig. 3C shows that the migration speed of the bands decreased progressively with an increasing number of strands in the assembly, with TDN and TDN-ssDNA exhibiting slower migration speed compared to other combinations, thereby confirming the successful assembly of the TDN framework structure. In addition, TCEP is crucial for keeping sulfhydryl groups in the reduced state by reducing disulfide bonds. The inclusion of TCEP at a final concentration ranging from 200 μM to 1 mM prior to heat denaturation effectively reduces the interference of sulfhydryl oxidation during the assembly process (Fig. S4A). However, as shown in Fig. S4A (lane 8), TCEP concentrations exceeding 3 mM may lead to TDN degradation. Moreover, adding TCEP post-assembly to sulfhydryl-modified TDNs did not yield markedly improvements (Fig. S4B). Additionally, Fig. S4C confirmed that the single-stranded terminus of TDN-ssDNA can be nonspecifically cleaved by AsCas12a.

In order to enhance the sensor performance, especially in terms of sensitivity, specificity, biocompatibility, and the comprehensive functionalization of metal nanoparticles, CFAu was utilized, in which the substantial specific surface area and biocompatibility facilitate the efficient immobilization of nucleic acid probes and expedited electron transfer between the nucleic acid bases and the electrode surface. Moreover, as a versatile carrier, the optimization and quantification of surface-modified functional groups can be readily achieved by tailoring the size, shape, and surface properties of the nanoparticles to accommodate a high payload of electroactive markers [32,33]. The morphology of the modified CFAu nanoparticles on the Au electrode surface was clearly visible through scanning electron microscopy (SEM), as depicted in Fig. 3D and Fig. S5. The main skeleton and side branches of CFAu provide superior electrical conductivity and an abundance of sulfhydryl binding sites for TDN-ssDNA. Additionally, its expansive specific surface area considerably enhances the attachment of Ru(bpy)_3_^2+^ after forming a monolayer of MPA on the CFAu surface, thereby markedly improving the performance of the electrochemical biosensor [34]. In cyclic voltammetry (CV) analysis, the augmented surface area of the CFAu-modified electrode was evidenced by the elevated peak current (Fig. 3E and Fig. S6A), as predicted by the Randle–Sevcik equation:

$$i_{p}=2.69\times 10^{5}ACn^{3/2}D^{1/2}v^{1/2}$$ (1)

where *i*_p_ denotes the peak current, *A* represents the electrode surface area, *C* is the volumetric concentration of the analyte, *n* indicates the electron transfer number, *D* refers to the diffusion coefficient of the analyte, and *v* is the scan rate. Among these parameters, with other factors held constant, an increase in the peak current (*i*_p_) suggests an enhancement in the electroactive area (*A*) [35]. Additionally, the electrochemical impedance spectroscopy (EIS) analysis revealed a decreased electron transfer resistance (*R*_et_), inversely related to the semicircle radius on the *Z*_re_ axis, as shown in Fig. 3F and Fig. S6B, indicating an enhanced electron transport capability. These findings collectively demonstrated the improved performance of the CFAu-modified electrode, aligning with the anticipated mechanism of the electrochemical biosensor. Sequential modification of the electrode with tetrahedral probes TDN-ssDNA and MPA led to restricted diffusion of the electronic medium at the electrode interface, resulting in a marked reduction in the peak of redox current and an increase in impedance, as confirmed in Fig. 3E and F and Fig. S6C to F, whose results support the biosensor’s development in line with the predicted mechanism.

### Specific detection of *fadA* by fluorescence analysis

**Fig. 4.**
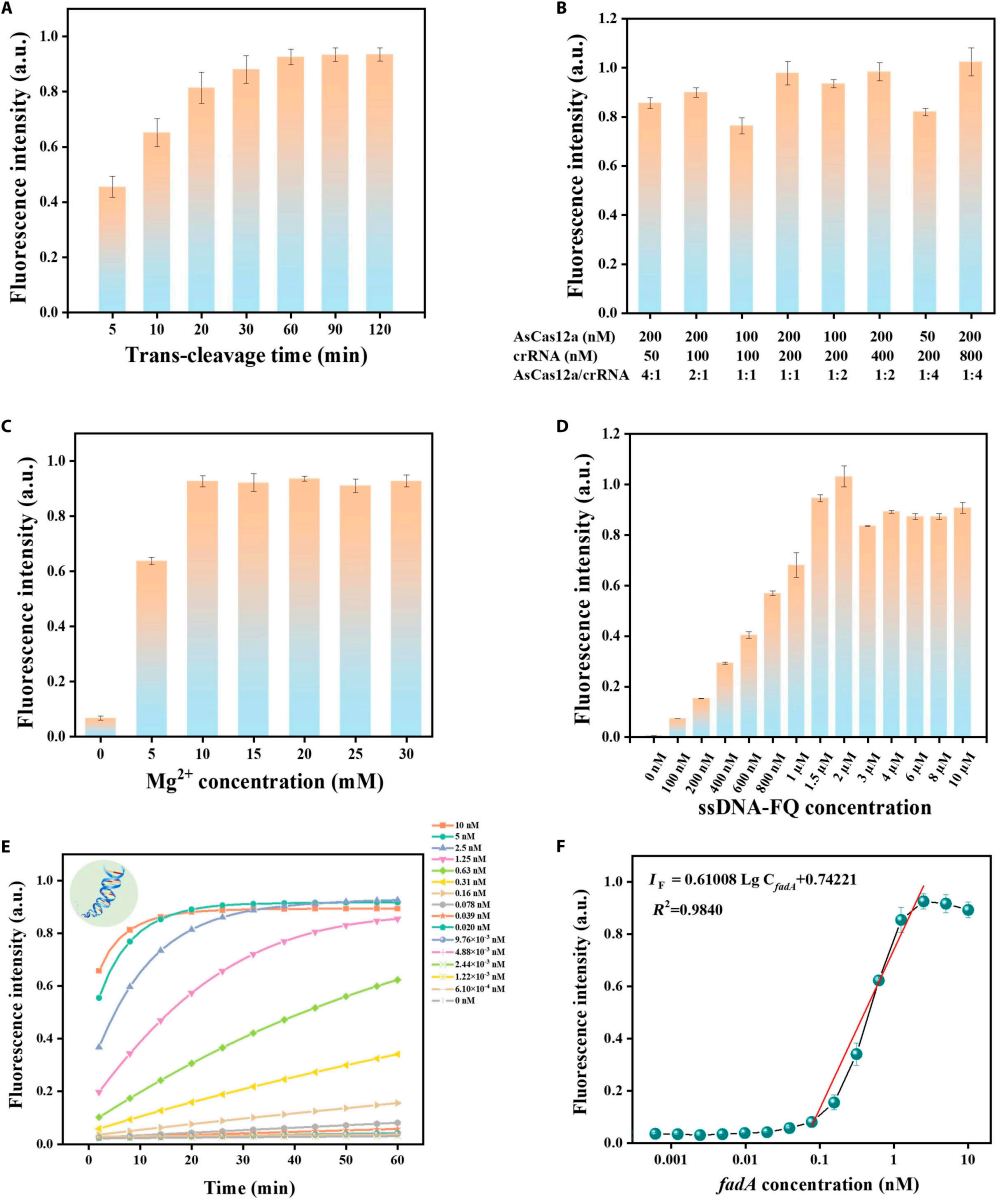
Optimization of detection conditions and specific detection of *fadA* by fluorescence analysis. (A) Optimization of AsCas12a/crRNA trans-cleavage duration. (B) Impact assessment of AsCas12a/crRNA ratios on enzymatic activity. (C) Evaluation of Mg^2+^ concentration effects on detection signals. (D) Assessment of ssDNA-FQ concentration effects on the fluorescence signal. (E) Fluorescence intensity of a series of *fadA* concentrations (6.10 × 10^−4^ to 10 nM) using previously optimized conditions: 60-min trans-cleavage time, AsCas12a/crRNA ratio = 100 nM:200 nM, Mg^2+^ concentration = 10 mM, ssDNA-FQ concentration = 1.5 μM. (F) Calibration curve of fluorescence intensity obtained from (E) versus *fadA* concentration. Error bars indicate mean ± SD, where *n* = 3 replicates.

The CRISPR/Cas12a system, in conjunction with crRNA, specifically recognizes the target gene *fadA*, leading to the nonspecific cleavage of the fluorescent probe ssDNA-FQ and the generation of fluorescent signals [36]. Prior to the fluorescence analysis, adjustments in the trans-cleavage time, the ratio of AsCas12a to crRNA, and Mg^2+^ concentration can finely tune the activity of the CRISPR/Cas12a system to enhance the sensitivity of the assay markedly. Utilizing fluorescence intensity as a marker, Fig. 4A shows a continuous increase in intensity within 1 h, reaching a plateau at that point. To minimize the variability caused by slight differences in data acquisition times and maximize the sensitivity and resolution of detection, the time (1 h) at which the fluorescence approaches saturation was designated as the reaction time. The binding of AsCas12a to crRNA is crucial for specific target nucleic acid sequence recognition and is directly linked to enzymatic activity and cleavage efficiency. Therefore, various concentrations and ratios of AsCas12a to crRNA have been incorporated into the CRISPR/Cas-based fluorescence assay, and the ratio of 100 nM:200 nM was determined after comprehensive consideration for subsequent fluorescence detection and ECL analysis (Fig. 4B). Meanwhile, Mg^2+^ concentration substantially influences the RuvC domain’s active pocket in AsCas12a. The bimetallic ion mechanism in the RuvC domain, facilitated by Mg^2+^ ions, induces conformational changes that enhance ssDNA’s spatial coordination at the active cleavage site [37]. Additionally, Mg^2+^ enhances Cas12a’s affinity for crRNA [38]. The effect of Mg^2+^ concentration on the trans-cleavage efficiency of the CRISPR/Cas12a system was thoroughly investigated, establishing 10 mM as the optimal Mg^2+^ concentration for stable fluorescence intensity (Fig. 4C). Additionally, upon trans-cleavage by activated AsCas12a/crRNA, the ssDNA in the fluorescent probe ssDNA-FQ displaces the quenching group (BHQ1) from the fluorophore (FAM), resulting in fluorescence emission. The concentration of ssDNA-FQ within the reaction system determines the saturation fluorescence intensity, which in turn affects the sensitivity of fluorescence detection. As depicted in Fig. 4D, increasing ssDNA-FQ concentration from 100 nM to 2 μM led to progressively higher fluorescence intensity. However, further increases in ssDNA-FQ concentration reduced the intensity, likely due to self-quenching at excessively high concentrations. After a thorough evaluation, a concentration of 1.5 μM ssDNA-FQ was chosen as optimal.

To evaluate the analytical performance of the AsCas12a/crRNA system, the *F. nucleatum*-specific gene *fadA* was serially diluted to act as an activator for AsCas12a/crRNA, while sterile water was employed as a negative control. Fig. 4E illustrates that fluorescence intensity escalated with the increase of *fadA* concentrations. Under optimal detection conditions, a strong linear correlation was found between *fadA* concentration and fluorescence intensity ranging from 0.078 to 2.5 nM *fadA*, with a calibration curve as $I_{F}=0.61008\;\mathrm{Lg}C\;\left(\mathrm{nM}\right)+0.74221\;\left(R^{2}=0.9840\right)$ (Fig. 4F).

### Sensitivity performance of the ECL biosensor

**Fig. 5.**
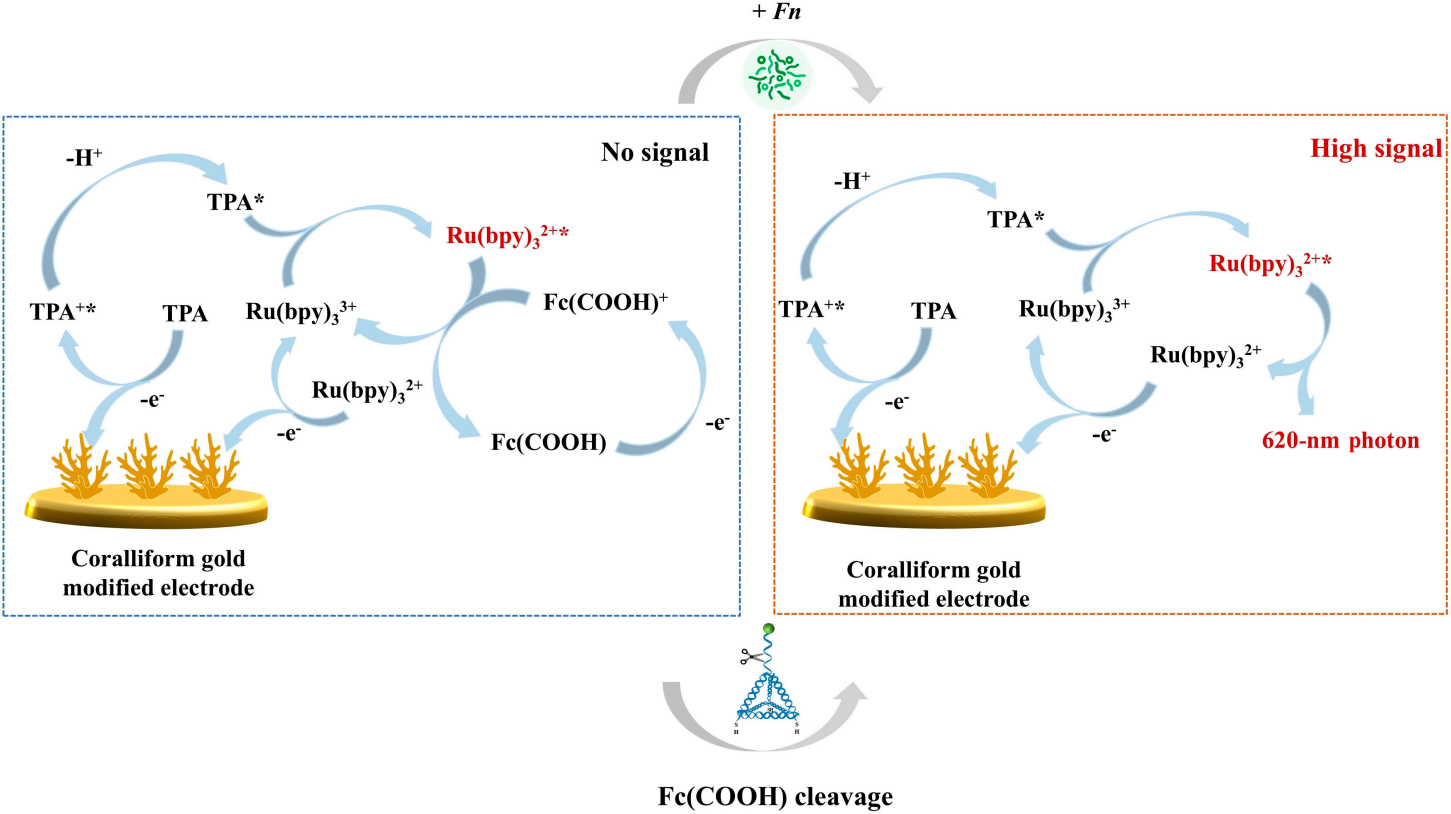
ECL mechanism of prepared CRISPR/Cas12a-based ECL biosensor.

**Fig. 6.**
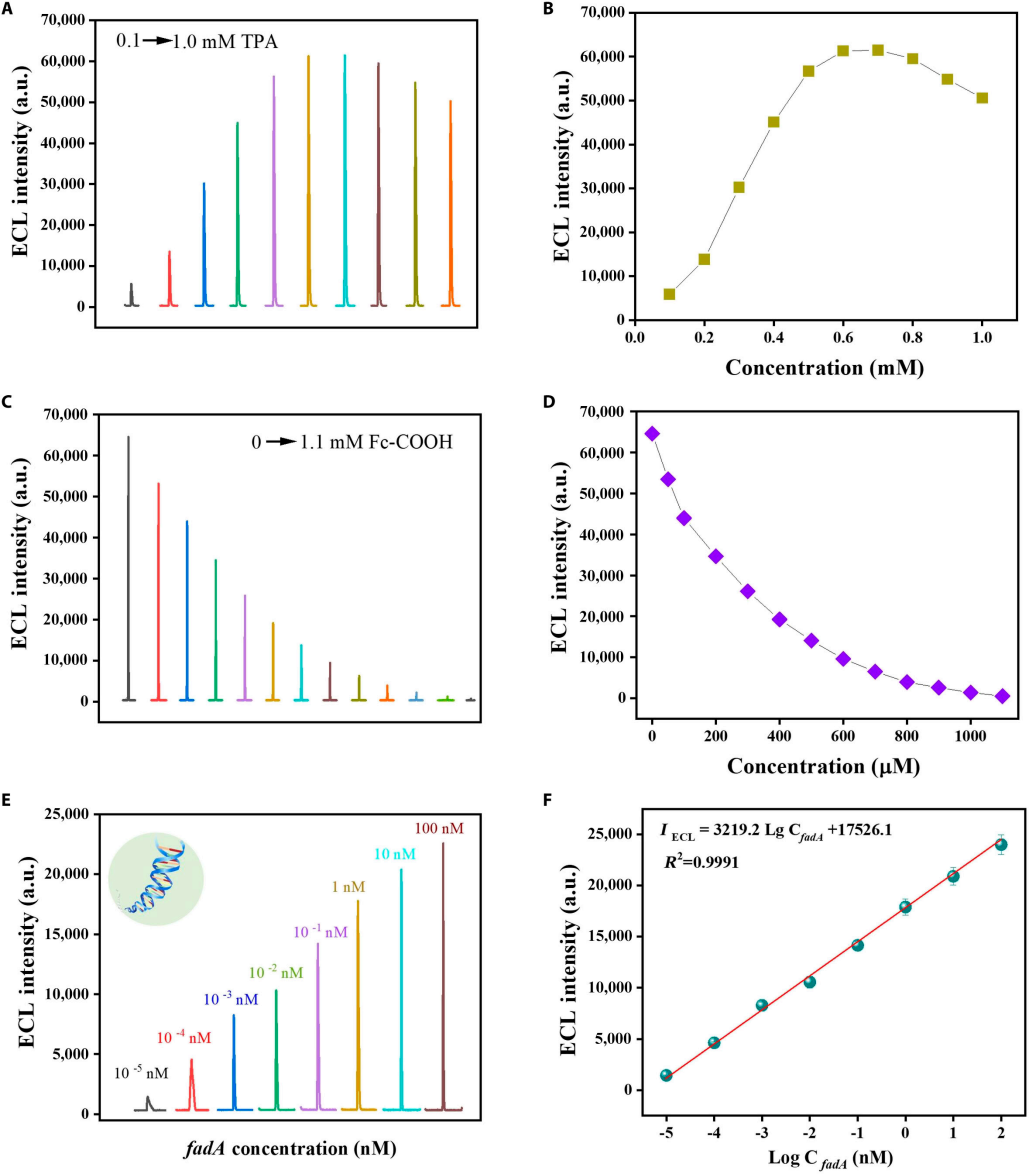
Optimization of ECL detection conditions and sensitive detection of *fadA* by ECL biosensor. (A) Evaluation of tripropylamine (TPA) concentration effects on ECL intensity. (B) ECL intensity variation with TPA concentration. (C) Assessment of ferrocene carboxylic acid (Fc-COOH) concentration effects on ECL intensity. (D) ECL intensity variation with Fc-COOH concentration. (E) ECL intensity response to varying *fadA* concentrations (10^−5^ to 100 nM) under optimized conditions: TPA concentration = 0.7 mM, Fc-COOH concentration = 1.1 mM. (F) Calibration curve of ECL intensity versus *fadA* concentration derived from (E). Error bars indicate mean ± SD, where *n* = 3 replicates.

Compared with traditional assays, as electrochemical reactions occur at the electrode surface, ECL reactions offer precise control over the emission location. Thus, the emission location is consistently on the electrode surface, boosting detection sensitivity, selectivity, the accuracy of multi-reagent detection, and imaging analysis. For ECL assays, the Ru(bpy)_3_^2+^-tripropylamine (TPA) co-reaction system stands out due to its high fluorescence quantum yield and remarkable stability [39]. Additionally, ferrocene (Fc) and its derivatives are recognized as effective quenching agents for Ru(bpy)_3_^2+^ [26]. The detailed luminescence and quenching mechanism were discussed in Fig. 5 and Supplementary Materials, which involves Ru(bpy)_3_^2+^ and TPA losing electrons at the electrode surface, forming Ru(bpy)_3_^3+^ and excited-state TPA*, respectively. These 2 species then react to form the excited-state Ru(bpy)_3_^2+^*. When Ru(bpy)_3_^2+^* returns to the ground state, it emits ECL at approximately 620 nm. However, the introduce of Fc can interact with Ru(bpy)_3_^2+^* in the excited state, effectively reducing ECL emission [26]. Herein, Fc-COOH can be bonded to the end of TDN-ssDNA via an amide bond, reducing the light emitted by Ru(bpy)_3_^2+^ on the electrode surface. As the concentration of incubated Fc-COOH increases, more binds to the electrode surface, further reducing the ECL background signal before trans-cleavage. As shown in Fig. 6A and B, in order to optimize the TPA concentrations, extensive testing has shown that ECL intensity peaks when the TPA concentration reaches 0.7 mM, establishing this as the optimal concentration. When the concentration of TPA is excessively high, it may alter the ionic strength of the solution, affecting the charge transfer efficiency and reducing the generation of excited-state Ru(bpy)₃^2+^*, which in turn diminishes the luminescent signal. Additionally, high concentrations of TPA can potentially trigger side reactions, altering the chemical environment and interfering with the normal ECL reaction, ultimately leading to a decrease in the detection signal. As shown in Fig. 6C and D, at a concentration of 1.1 mM Fc-COOH, the quenching efficiency neared 99%, making 1.1 mM the optimal concentration for Fc-COOH.

After the successful preparation of CRISPR/Cas12a-based ECL biosensor, to highlight the superiority of TDN-ssDNA as a recognition unit scaffold for linking the quenching agent Fc-COOH, a comparative study was conducted using ssDNA modified with sulfhydryl and amino groups at both ends. The 1-nM *fadA* gene was targeted for the AsCas12a/crRNA-based ECL analysis with/without the modification of TDNs. Fig. S7A and B shows that the TDN-ssDNA-modified electrode exhibited approximately 2-fold higher ECL signal than the ssDNA-modified electrode. This marked enhancement is attributed to the high precision density and orientation of the terminal ssDNA probes provided by TDN-ssDNA, which greatly improves the efficiency of recognition and cleavage by AsCas12a/crRNA.

For the sensitive detection of *fadA,* as depicted in Fig. 6E and F, the ECL intensity substantially increased with a gradual increase in *fadA* concentration, ranging from 10^−5^ to 100 nM. This augmentation in ECL intensity was accompanied by a robust linear relationship between the logarithm of *fadA* concentration and the ECL intensity, as described by the correlation equation $I_{\mathrm{ECL}}=3,219.22\;\mathrm{Lg}C\;\left(\mathrm{nM}\right)+17,526.1\;\left(R^{2}=0.9991\right)$. Compared to mentioned results using fluorescent methods (Table S2), the ECL approach offered a broader detection range and higher sensitivity for both *fadA* and *F. nucleatum* assays, because ECL reactions can mitigate the instabilities seen in fluorescent reactions due to unstable reagents, thereby improving the controllability of these reactions, which potentially reduced the limit of detection (LOD) for *fadA* by 3 orders of magnitude to the femtomolar level.

### Ultrasensitive detection of *F. nucleatum* with single colony by amplification-free ECL biosensor

**Fig. 7.**
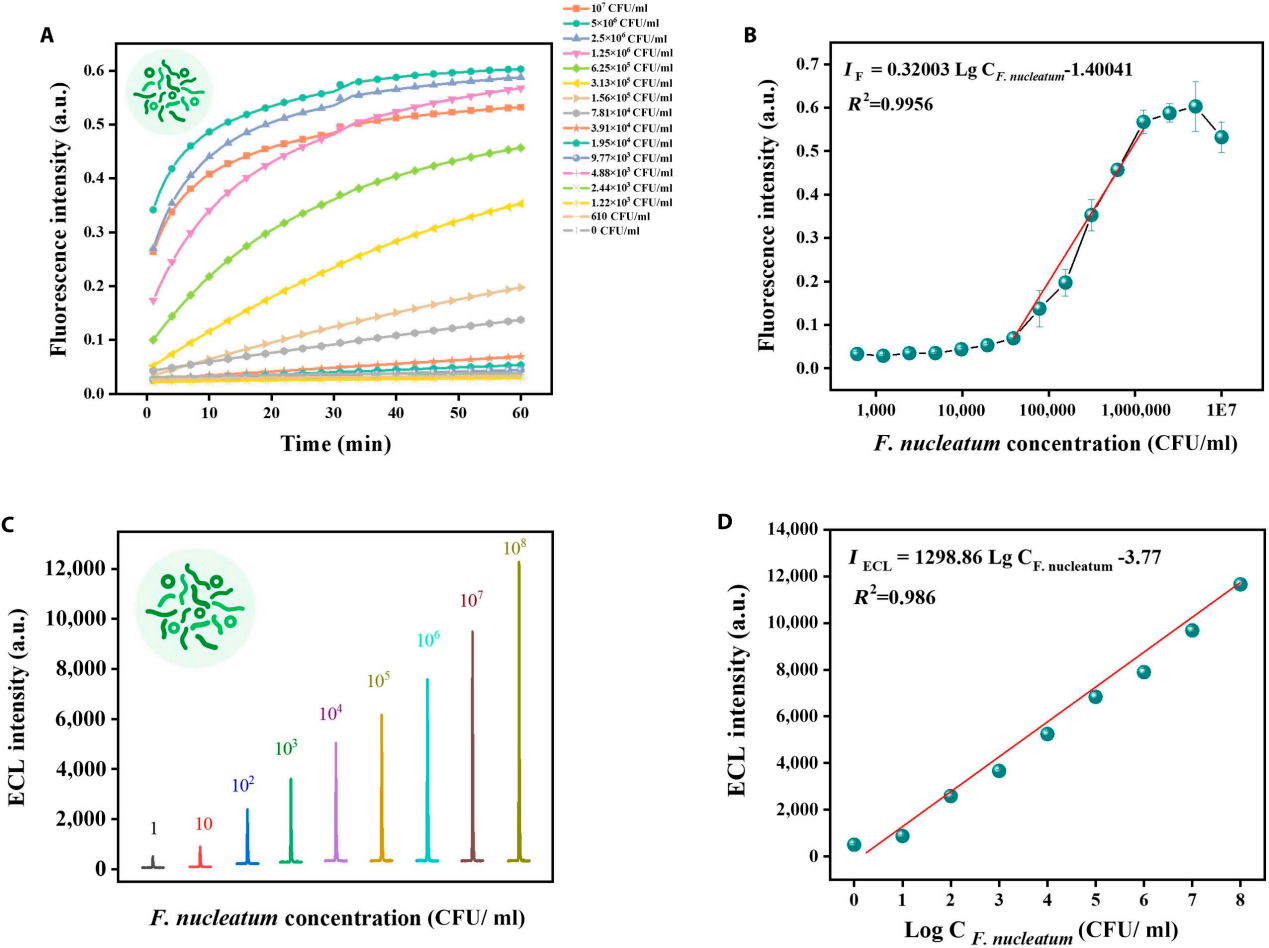
Detection performance of the CRISPR/Cas12a-based fluorescence analysis and ECL biosensor. (A) Fluorescence intensity of a series of *F. nucleatum* concentrations (610 to 10^7^ CFU/ml). (B) Calibration curve of fluorescence intensity obtained from (A) versus *F. nucleatum* concentration. (C) ECL intensity response to varying *F. nucleatum* concentrations (1 to 10^8^ CFU/ml). (D) Calibration curve of ECL intensity versus *F. nucleatum* concentration derived from (C). Error bars indicate mean ± SD, where *n* = 3 replicates.

Furthermore, to verify whether AsCas12a/crRNA can detect the *fadA* gene within the genome and trigger trans-cleavage activity in actual bacterial assays, we performed fluorescence analysis using different concentrations of *F. nucleatum* genomic extracts. As depicted in Fig. 7A, fluorescence intensity progressively increased with higher concentrations of *F. nucleatum*. Additionally, a strong linear relationship was observed between fluorescence intensity and the logarithm of *F. nucleatum* concentration, from 39,063 to 1,250,000 CFU/ml with a calibration curve of $I_{F}=0.32003\;\mathrm{Lg}C\;\left(\mathrm{CFU}/\mathrm{ml}\right)-1.40041\;\left(R^{2}=0.9956\right)$ (Fig. 7B). Similarly, for the ECL detection of *F. nucleatum*, in the case of progressive amount of *F. nucleatum*, the ECL intensity increased dramatically from 581.5 arbitrary units (in 1 CFU/ml *F. nucleatum*) to 12,363.1 arbitrary units (in 10^8^ CFU/ml *F. nucleatum*), as shown in Fig. 7C. The ECL intensity demonstrated a linear correlation with the logarithm of *F. nucleatum* concentration, as represented by the equation $I_{\mathrm{ECL}}=1,298.86\;\mathrm{Lg}C\;\left(\mathrm{CFU}/\mathrm{ml}\right)-3.77$, with an *R*^2^ value of 0.986 (Fig. 7D).

Additionally, the sensing performance of the proposed biosensor was compared with contemporary electrochemical analysis techniques utilizing the CRISPR/Cas system in Table S3, indicating that our strategy achieves a relatively low detection limit and a broad linear range without amplification, thereby streamlining the detection process and reducing the overall time required for analysis. Moreover, the ECL sensor’s performance was benchmarked against other published detection methods of *F. nucleatum* in Table 1. Notably, the sensitivity of the CRISPR/Cas12a-based ECL detection strategy surpassed that of previously reported rapid detection methods for *fadA*, such as quantitative colorimetric loop-mediated isothermal amplification technique using the phenol red indicator (QLAMP-PhR) (206 copies/μl), recombinase polymerase amplification (RPA) and CRISPR/Cas12a-based fluorescence assay, and lateral flow immunoassay (LOD = 5 copies/μl) [40,41]. Furthermore, while combining culture and RNA-cleaving fluorogenic DNAzyme (RFD) can detect *F. nucleatum* as low as 1 CFU/ml, it necessitated a prolonged culturing period of 36 h, which substantially impeded detection efficiency. Herein, the amplification-free ECL biosensor based on CRISPR/Cas12a achieved ultra-low detection at the single-colony level and offered a wide detection range from 1 to 10^8^ CFU/ml of *F. nucleatum*, making it sensitive enough for early CRC diagnosis.

**Table 1.** A comparison of different methods for detecting *Fusobacterium nucleatum*

Year	Method	Target	Assay time	Detection range	LOD	Ref.
2016	LAMP	*fadA*	60 min	-	225 pg/ml (9.95 × 10^5^ fM)	[42]
2020	RCA-based fluorescence quenching-recovery sensor	*F. nucleatum*	170 min	0.7–70,000 pg/ml (288–2.88 × 10^7^ CFU/ml)	0.7 pg/ml (288 CFU/ml)	[43]
2023	Culture and RFD	*F. nucleatum*	36 h	-	1 CFU/ml	[44]
2024	QLAMP-PhR	*fadA*	51 min	-	206 copies/μl	[40]
2024	RPA-CRISPR-Cas12a (fluorescent detection system and lateral flow immunoassay)	*nusG*	30–40 min	-	5 copies/μl	[41]
2025	CRISPR/Cas12a-based fluorescence detection	fadA and *F. nucleatum*	105 min	0.078–2.5 nM 39,063–1,250,000 CFU/ml	0.078 nM 39,063 CFU/ml	This work
2025	CRISPR/Cas12a-based ECL	*fadA* and *F. nucleatum*	105 min	10^−5^–100 nM 1–10^8^ CFU/ml	10^−5^ nM 1 CFU/ml	This work

LAMP, loop-mediated isothermal amplification; RCA, rolling circle amplification; RFD, RNA-cleaving fluorogenic DNAzyme; QLAMP-PhR, quantitative colorimetric loop-mediated isothermal amplification technique using the phenol red indicator; RPA, recombinase polymerase amplification; ECL, electrochemical luminescence

### Stability and selectivity of amplification-free ECL biosensor

**Fig. 8.**
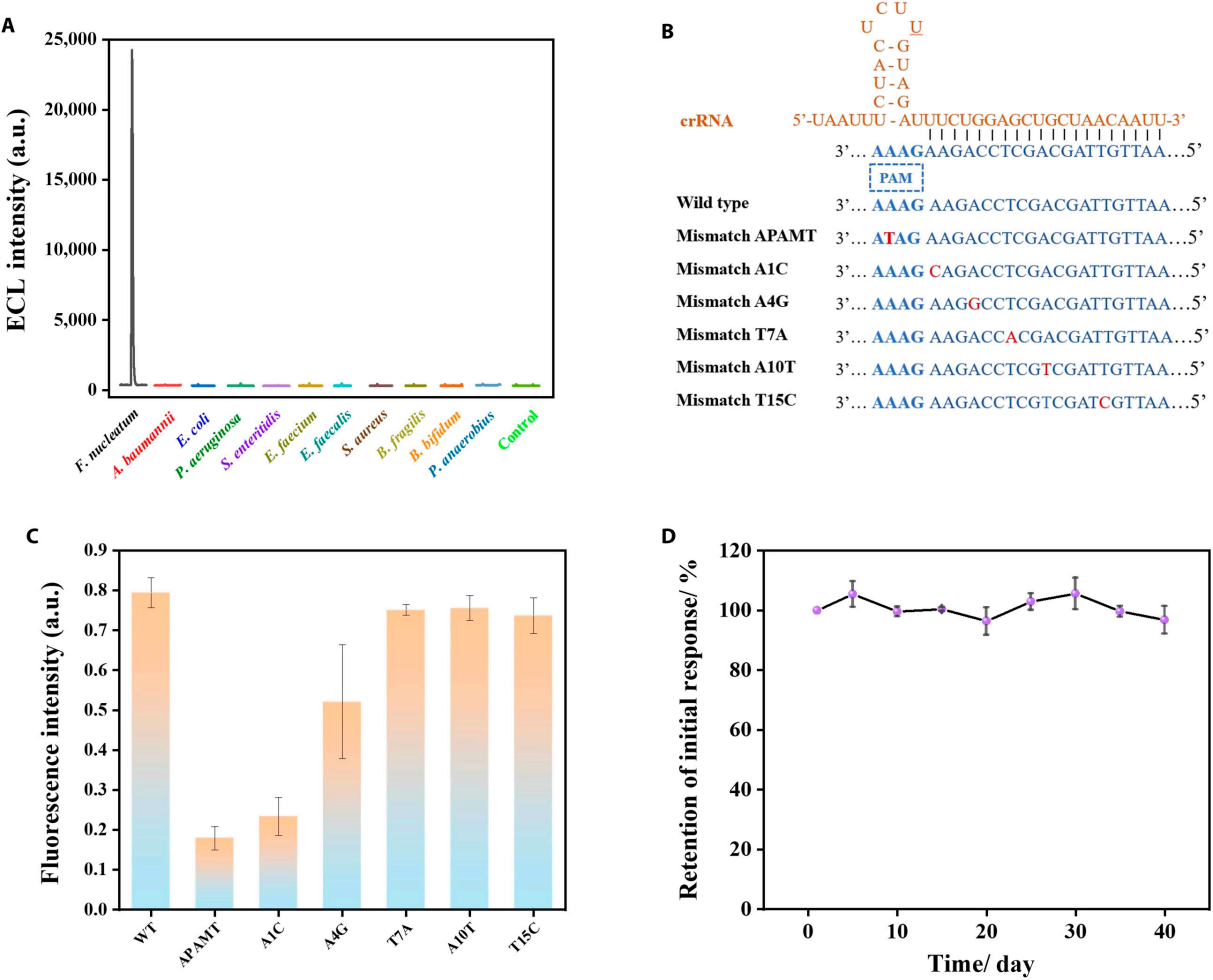
Stability and selectivity of the ECL biosensor. (A) Specificity analysis of the CRISPR/Cas12a system for *F. nucleatum* versus other bacteria by ECL. (B) Design of target strands with point mutations or mismatches at different positions. (C) Evaluation of the effect of point mutations or mismatches at different positions on CRISPR/Cas12a system activity by fluorescence assay. (D) Stability assessment of ECL sensors for *fadA* analysis over 40 d. Error bars indicate mean ± SD, where *n* = 3 replicates.

In the practical application of sample detection, a robust analytical method must distinguish *F. nucleatum* from the diverse bacterial communities present in its surrounding environment. To evaluate the specificity of the CRISPR platform, 10 strains including *Acinetobacter baumannii*, *Escherichia coli*, *Pseudomonas aeruginosa*, and *Enterococcus faecalis*, among others, were analyzed using both fluorescence and ECL methods (Fig. 8A and Fig. S8). The results indicated that only the *F. nucleatum* exhibited strong fluorescence intensity and ECL signal, whereas the signals from other bacteria were similar to those of the control group. These findings conclusively demonstrate that, in addition to efficient signal amplification by AsCas12a and flexible crRNA programmability, the constructed CRISPR/Cas12a system possesses exceptional species-specific selectivity, which is essential for accurately and specifically recognizing *F. nucleatum* within the complex stool environment.

The mechanism by which AsCas12a/crRNA identifies target genes was previously discussed, i.e., sequence matching in the PAM-proximal region is pivotal. Herein, the CRISPR/Cas system may exhibit heightened sensitivity to single-nucleotide mismatches in this region. As shown in Fig. 8B, TSs with single-point mutations or mismatches at various positions were designed (denoted as Mismatch APAMT, Mismatch A1C, Mismatch A4G, Mismatch T7A, Mismatch A10T, and Mismatch T15C) and tested using the fluorescence detection system at a final concentration of 10 nM. Fig. 8C illustrates that AsCas12a/crRNA exhibited enhanced sensitivity to mismatches near the PAM region, while mutations further from the PAM region had greater fault tolerance, which supports earlier findings that the PAM sequence is crucial for activating Cas12a enzymatic activity and that the CRISPR/Cas12a-based nucleic acid detection system has a high resolution for detecting single-nucleotide variations near PAM sequences [45,46]. As single-nucleotide mutations (e.g., in *EGFR* and *KRAS* genes) are often linked to the onset and progression of the disease and drug sensitivity [47–49], distinguishing between wild-type and mutant sequences is pivotal for disease diagnosis and treatment decision-making in practical clinical applications. Accurately differentiating single-point mutations by biosensors can effectively avoid false-negative or false-positive results. Our prepared CRISPR/Cas12a-based biosensors achieve precise typing by distinguishing between wild-type and mutant sequences through leverage of the strict complementarity between crRNA and the target sequence. Furthermore, this method can detect mutations without prior knowledge of target sequence concentration [46], proving useful in genotype screening and enhancing genetic analysis precision.

On the other hand, the long-term storage stability of in vitro diagnostic devices under optimal conditions is of paramount importance. To assess this, a substantial number of CFAu/TDN-Fc/MPA/Ru(bpy)_3_^2+^-modified electrodes were fabricated and stored in phosphate-buffered saline (PBS) at 4 °C to evaluate the biosensor’s storage stability. As depicted in Fig. 8D, ECL data for *fadA* were collected at 5-d intervals over a period of 40 d. The results showed only minor fluctuations within less than 10%, possibly due to partial instability, detachment, or electrode surface material decomposition, demonstrating that the stability of the sensing platform is satisfactory and holds the potential for in vitro CRC diagnosis.

## Conclusion

In this study, the CRISPR/Cas12a system was developed for the selective detection of *fadA*, a specific gene in *F. nucleatum*, followed by the verification of fluorescence and ECL analysis. The prepared CRISPR/Cas12a-based ECL biosensor leverages the signal amplification effect of AsCas12a enzyme during activation, the programmability of crRNA, and the specificity of AsCas12a/crRNA in target recognition, achieving ultrasensitive detection of even single-colony *F. nucleatum* without amplification. Specifically, the prepared biosensor, modified with CFAu, offers superior conductivity and abundant thiol binding sites, while the increased surface area greatly facilitates the soft landing of the luminescent agent Ru(bpy)_3_^2+^, achieving dual signal amplification on the basis of AsCas12a. Moreover, the introduction of TDN-ssDNA with a rigid structure as a special scaffold not only serves as a binding site for the quencher Fc-COOH but also provides precise control over the density and orientation of the ssDNA probe at its terminus, enhancing the accessibility and reactivity of the AuCas12a enzyme. Consequently, the developed ECL biosensor achieves gene detection at the femtomolar level without gene amplification. Hence, our ECL platform outperforms conventional fluorescence detection methods with a detection range of 10 fM to 100 nM for *fadA* and 1 to 10^8^ CFU/ml for *F. nucleatum* under optimal conditions. Furthermore, the AsCas12a/crRNA-based ECL biosensor exhibits high selectivity and excellent stability, facilitating the precise detection of *F. nucleatum* in complex environmental samples and holding potential for CRC in vitro diagnostics. Moreover, the high mismatch sensitivity of AsCas12a to PAM and nearby regions offers a robust strategy for distinguishing mutant from wild-type sequences, allowing mutation detection without prior target sequence concentration knowledge, thus enhancing genotype screening and genetic analysis precision. In conclusion, the CRISPR/Cas12a-based ECL platform developed in this study demonstrates high sensitivity, selectivity, and stability in quantitatively detecting *F. nucleatum*. This biosensor is anticipated to be further refined for clinical analysis of actual fecal samples in the near future.

Moreover, the electrochemical sensing platform based on the CRISPR/Cas system serves as a reliable detection method for *F. nucleatum* and can be tailored for detecting other nucleic acids and pathogens by rationally designing crRNA sequences. Additionally, by means of microfluidic technology and programming of crRNA, multiple markers can be detected simultaneously, thereby enhancing the accuracy of disease diagnosis [50,51]. Overall, this method can be applied to biosensing platforms for various disease biomarkers, thereby advancing the development of biosensors and improving the early diagnosis system for diseases.

## Data Availability

The data supporting this study’s findings are available from the corresponding author upon reasonable request.

## Supplementary Materials

Supplementary 1 Supplementary Text Figs. S1 to S8 Tables S1 to S3 References
