# Supplementary material for: Amplification-Free Electrochemiluminescent Biosensor for Ultrasensitive Detection of Fusobacterium nucleatum Using Tetrahedral DNA-Based CRISPR/Cas12a
Source: Cyborg Bionic Syst. 2025 May 1;6:0266. doi: 10.34133/cbsystems.0266 (PMC12044220; doi:10.34133/cbsystems.0266)
Supplement: Supplementary 1 — Supplementary Text Figs. S1 to S8 Tables S1 to S3 References [file cbsystems.0266.f1.docx]

**Supporting Information**

**Amplification-free electrochemiluminescent biosensor for ultrasensitive detection of *Fusobacterium nucleatum* using tetrahedral DNA based CRISPR/Cas12a**

Xindan Zhang^1,2^, Minkang Wu^2^, Haoran Shi^3^, Soochan Kim^4^, Shixiang Lu^1,*^, Ping Wang^2,*^, Jieling Qin^1,2,^^*^

1. School of Chemistry and Chemical Engineering, Beijing Institute of Technology Zhengzhou Academy of Intelligent Technology, Beijing Institute of Technology, Beijing 100081, China
2. Tongji University Cancer Center, Shanghai Tenth People's Hospital, School of Medicine, Tongji University, Shanghai 200092, China
3. School of Bioengineering, Dalian University of Technology, Dalian City, Liaoning 116024, China
4. School of Chemical Engineering, Sungkyunkwan University, South Korea

* Correspondence: qinjieling770@hotmail.com (Jieling Qin), wangp@tongji.edu.cn (Ping Wang) and shixianglu@bit.edu.cn (Shixiang Lu)

**Materials**

EnGen® LbaCas12a was purchased from New England Biolabs Ltd. (New England, USA) for comparison. The four single strands of tetrahedral DNA nanostructures, ssDNA, and crRNA were designed with the detailed sequence in **Table S1** and synthesized by Sangon Biotech (Shanghai) Co., Ltd. (Shanghai, China), the ssDNA-FQ reporter was synthesized by Tsingke Biotechnology Co., Ltd. (Beijing, China). Chemicals such as 3-mercapto propionic acid (MPA), N-hydroxy-succinimide (NHS), N-(3-dimethyl aminopropyl)-N’-ethylcarbodiimide hydrochloride (EDC), morpholino ethanesulfonic acid (MES), K_3_Fe(CN)_6_ and K_4_Fe(CN)_6_ were purchased from Sigma-Aldrich Co., Ltd. (Shanghai, China). Tris-HCl, EDTA, NaOH, H_2_SO_4_, MgCl_2_, NaCl, and KCl were obtained from Sangon Biotech (Shanghai) Co., Ltd. (Shanghai, China) and used without further purification. Tris (2,2-Bipyricy) Dichlororuthenium (ll) Hexahvdratel, Carboxyferrocenel, N, N-Dipropy-1-Propanamine, chloroauric acid hydrate, and tripropylamine were received from Adamas.

**Characterizations**

All electrochemical and electrochemiluminescence measurements were conducted using the LK5100Plus multifunctional ECL system from Lanlike (Tianjin) Chemical & Electronic High-Tech CO., Ltd., China, which included a traditional three-electrode cell comprising a modified gold electrode (diameter = 2 mm) as the working electrode, platinum foil as the counter electrode, and an Ag/AgCl electrode as the reference. The surface morphologies of the coralline gold electrode were characterized by scanning electron microscopy (SEM, JEOL JSM-700F, Japan). In cyclic voltammetry measurement, the conditions were constant as follows: applied potential 0 -0.5 V in 20 mM Fe (CN)_6_^3-/4-^/PBS (pH 7.4) at a scan rate of 10 mVs^-1^. Moreover, electrochemical impedance spectroscopy (EIS) with a sine wave of 10 mV amplitude together with the frequency range of 0.1 Hz to 100 kHz in 20 mM Fe (CN)_6_^3-/4-^/PBS (pH 7.4) solution followed by the simulation by ZSimpWin EIS DATA analysis software (Perkin-Elmer, Version 2.00) with an appropriate equivalent circuit. Moreover, for electrochemiluminescence detection, 5 uL of the above Cas12a/crRNA/*fadA* ternary complex was dropped onto the prepared sensor and incubated at 37°C for 60 min. ECL intensities were measured by applying cyclic voltammetry (CV) (100 mV/s, 0-1.4 V) in PBS solution with optimized conditions containing 0.7 mM TPA, as well as the attachment of 1.1 mM Fc-COOH concentration at the end of TDN-ssDNA.

**Acquisition of the *fadA* gene and extraction of genomic DNA from *F. nucleatum***

The *fadA* gene was amplified from the plasmid using polymerase chain reaction (PCR) with the high-fidelity KOD FX enzyme from TOYOBO Biotech CO., Ltd. (Shanghai, China). Specifically, the reaction mixture in a 96-well PCR machine included the plasmid, forward and reverse primers, 2×PCR buffer, dNTPs, and sterilized water, subjected to a 2-step cycle program. The obtained PCR products were afterward analyzed on 1% agarose gel and purified using the Universal DNA Purification Kit from TIANGEN Biotech Co., Ltd. (Beijing, China). Genomic DNA was extracted from a 1 mL bacterial culture suspension using the TIANamp Bacteria DNA Kit from TIANGEN Biotech Co., Ltd. (Beijing, China). The general procedure is as follows: Firstly, bacterial cells were collected from the culture by centrifugation and resuspended in Buffer GA. Subsequently, Proteinase K and Buffer GB were added, and the solution was incubated at 70°C until it became clear. Anhydrous ethanol was then added and mixed thoroughly. The solution along with any flocculent precipitate was transferred into a spin column CB3 and centrifuged. The column was washed sequentially with Buffer GD and Buffer PW, followed by complete drying. Finally, ddH_2_O was added dropwise onto the adsorption membrane, and the genomic DNA solution was collected by centrifugation. The DNA product should be stored at -20°C.

**Fluorescence analysis**

For fluorescence analysis, AsCas12a and crRNA were mixed in NEBuffer 2.1 and incubated at room temperature for 15 min to form Cas12a/crRNA structures. The *fadA* was then added, mixed, and incubated at 37°C for 30 minutes to create the Cas12a/crRNA/*fadA* complex. Subsequently, ssDNA-FQ was introduced to the mixture and incubated in a Roche lightcycler96 fluorescence quantitative PCR instrument at 37°C for 60 minutes. Fluorescence intensity was measured using a 470 nm excitation wavelength and 514 nm emission wavelength, monitoring the trans-cleavage activity per minute. After completion of the reaction, the system was placed under blue light for observation.

**Preparation of CFAu-based ECL sensor**

Before modification, electrodes were physically and chemically polished using Al_2_O_3_ (0.3um and 0.05um), piranha solution (30% H_2_O_2_: 18M H_2_SO_4_ = 1: 3, V: V) respectively, and then electrochemically cleaned in 0.5 M KOH and 0.5 M H_2_SO_4_ solution sequentially, followed by the electrochemical properties measurement in CV and electrochemical impedance spectroscopy (EIS) in a 20mM Fe(CN)_6_^3−/4−^ solution. CFAu was prepared by immersing the polished and N_2_-dried gold electrode in an aqueous solution of 0.1 M Na_2_SO_4_ and 50 mM HAuCl_4_ at -0.6 V for 500 s [1, 2]. The electrochemical properties of the electrochemically synthesized CFAu were investigated by CV and EIS. 3 μL of TDN-ssDNA was afterward dropped on the electrode surface in a buffer containing TCEP at room temperature for 3h. The successful coverage of TDN-ssDNA on the CFAu electrode was confirmed by CV and EIS. After washing with ddH_2_O and drying, the electrode was immersed in an MPA solution (dd water: EtOH = 3: 1, V: V) for 24h to form MPA SAM. The Ru(bpy)_3_^2+^ as the signal probe was then soft-landed on MPA, because of the charge retention by this organometallic dication on terminal carboxyl groups, resulting in covalent binding of a fraction of deposited Ru(bpy)_3_^2+^ molecules on the MPA SAM surface [3-5]. After that, Fc-COOH as a quenching probe was activated in a MES buffer containing EDC/NHS and bound to the end of TDN-ssDNA via amide bonds. The possible quenching processes of Fc-COOH on Ru(bpy)_3_^2+^ were as follows [6]:

| Fc-COOH - e^-^  Fc-COOH^+^ | 1 |
| --- | --- |
| Ru(bpy)_3_^2+^ - e^-^ Ru(bpy)_3_^3+^ | **2** |
| TPA - e^-^ TPA^+^* | **3** |
| TPA^+^* TPA*+ H^+^ | **4** |
| Ru (bpy)_3_^3+^ + TPA* Ru(bpy)_3_^2+^* +TPA | **5** |
| Fc-COOH^+^+ Ru(bpy)_3_^2+^* Fc-COOH+ Ru (bpy)_3_^3+^ | **6** |
| Ru(bpy)_3_^2+^* Ru(bpy)_3_^2+^*+ hυ | **7** |

For ECL detection of the target *fadA* gene, ECL signals were recorded by applying CV at 100 mV/s from 0 to 1.4 V in a PBS solution containing TPA. Firstly, in the absence of the target *fadA* gene, the oxidation of Fc-COOH leads to the formation of stable Fc-COOH^+^ at the terminus of TDN-ssDNA (Eq. 1). Meanwhile, the soft-landed Ru(bpy)_3_^2+^ is electrooxidized to Ru(bpy)_3_^3+^on the surface of ECL sensor (Eq. 2). With the introduction of TPA in the electrolyte, TPA releases electrons to form the cationic radical TPA (TPA^+*^) (Eq. 3), which rapidly loses a proton to become the tripropylamine radical TPA (TPA*) (Eq. 4). Subsequently, TPA*can react with Ru (bpy)_3_^3+^, further forming excited states Ru(bpy)_3_^2+^ (Ru(bpy)_3_^2+^*) (Eq. 5). However, the presence of the oxidized Fc-COOH^+^ afterwards interacts with Ru(bpy)_3_^2+^* inhibiting ECL emission (Eq. 6), and further quenching the system's ECL signals. Upon the addition of the target *fadA* gene, the AsCas12a/crRNA is activated with trans-cleavage activity, cutting the TDNs-linked ssDNA, thereby removing the ECL quencher Fc-COOH. Consequently, ECL emission at approximately 620 nm is produced and detected, marking the sensitive detection of the *fadA* gene as the excited states Ru(bpy)_3_^2+^* revert to the ground states Ru(bpy)_3_^2+^ (Eq. 7).


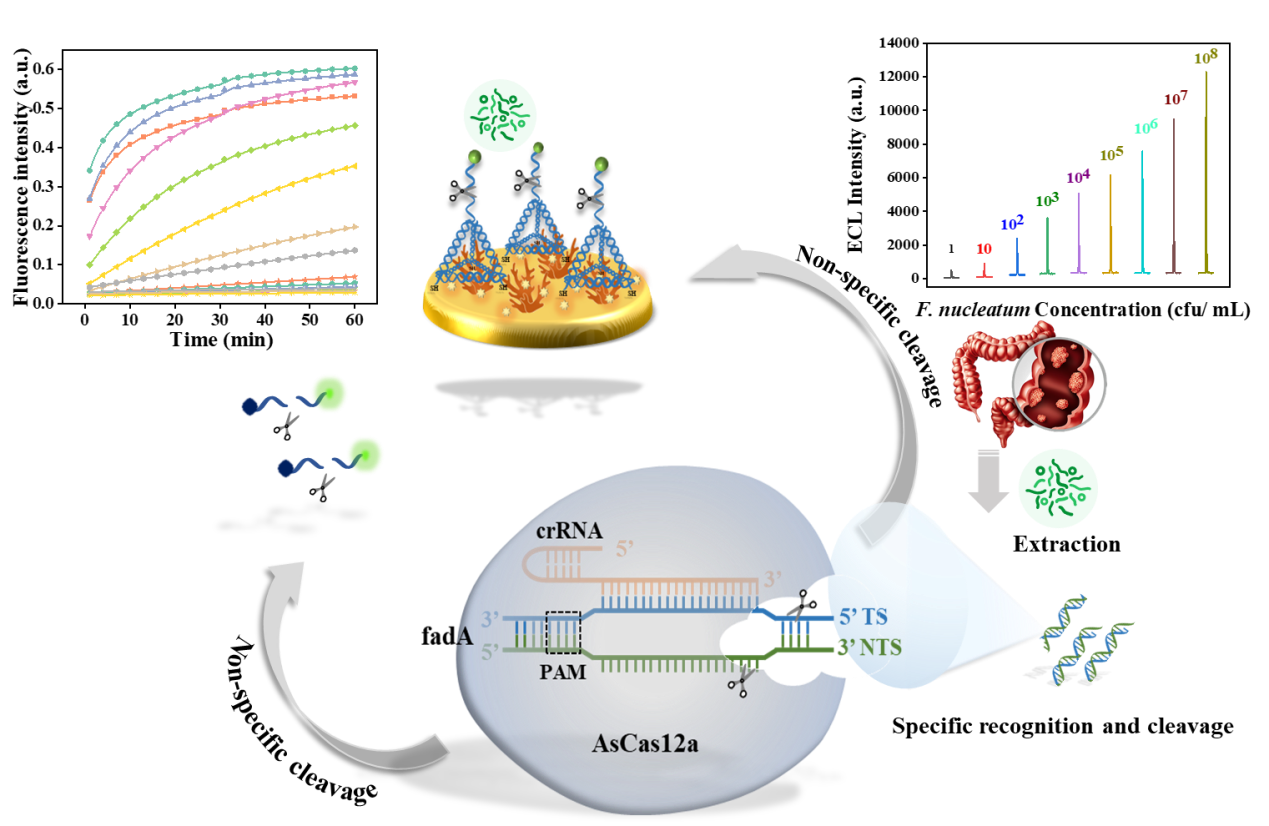


**Fig. S1.** Schematic illustration of an amplification-free electrochemical biosensor for sensitively detecting *F. nucleatum* based on the CRISPR/Cas system and tetrahedral DNA nanostructures (TDNs), while also using fluorescence for simple and rapid real-time monitoring.


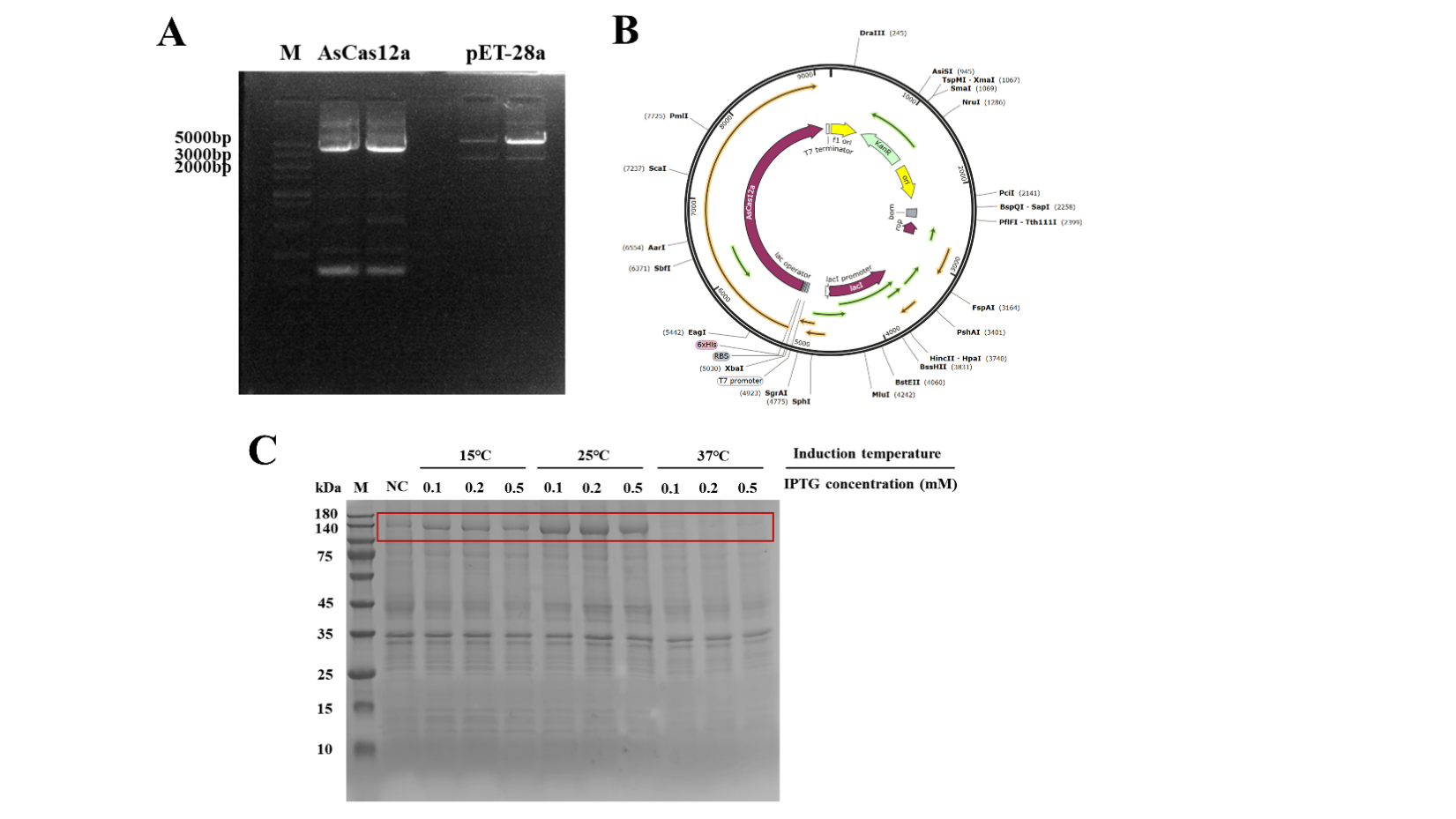


**Fig. S2.** Plasmid construction and expression condition testing. (A) Agarose gel electrophoresis of the pET28a vector and AsCas12a fragment generated by polymerase chain reaction (PCR). (B) Prokaryotic expression vector pET28a-AsCas12a with His tag. (C) Sodium dodecyl-sulfate polyacrylamide gel electrophoresis (SDS-PAGE) of the small-scale expression test of AsCas12a protein.


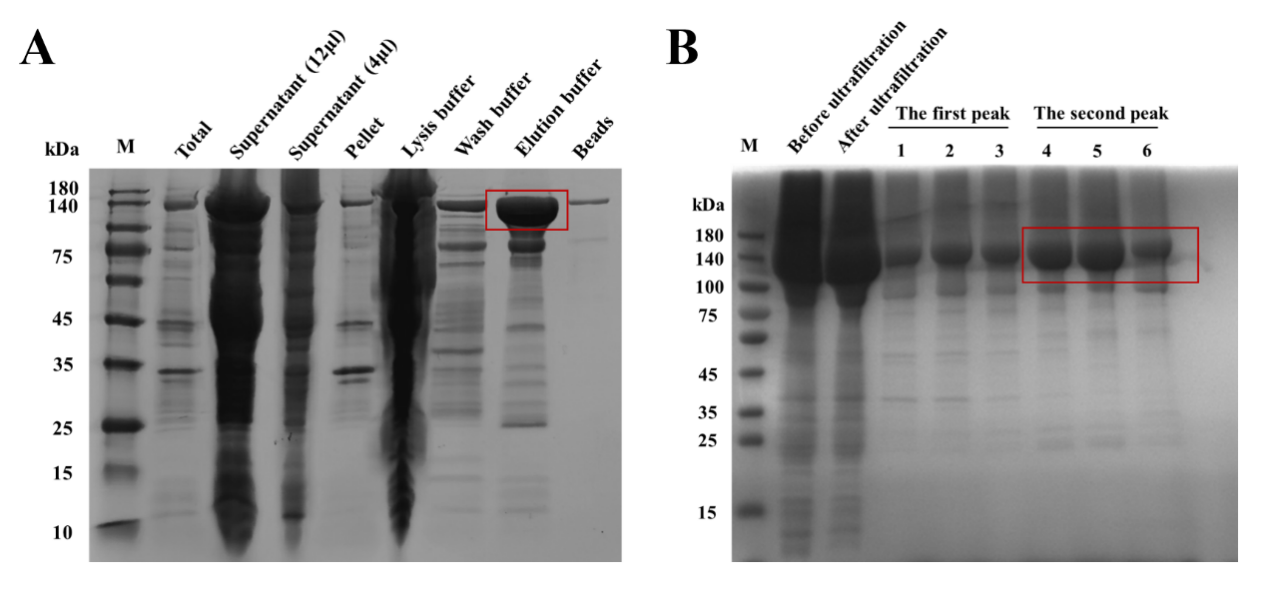


**Fig. S3.** Purification of AsCas12a enzyme. (A) SDS-PAGE analysis of AsCas12a purified using Ni-NTA beads. (B) SDS-PAGE analysis of AsCas12a following additional molecular sieving purification.


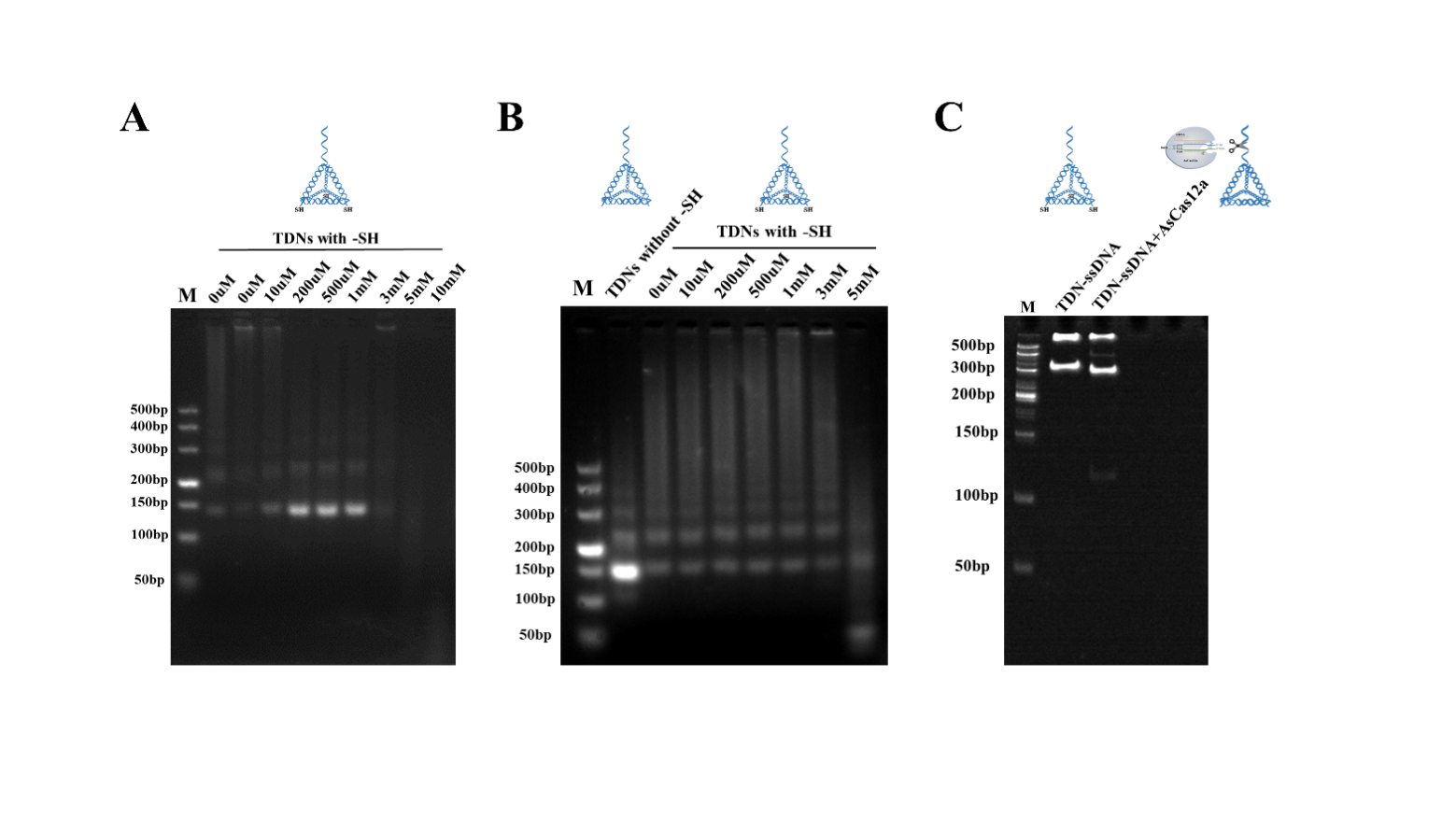


**Fig. S4.** Preparation and characterization of TDN-ssDNA. (A) 3% agarose gel electrophoresis of sulfhydryl-modified TDNs (TDNs with -SH) formed by adding different concentrations of TCEP prior to heat denaturation. (B) 3% agarose gel electrophoresis of TDNs with -SH formed by adding different concentrations of TCEP after the formation of TDNs, with TDNs without thiol modification (TDNs without -SH) as the control group. (C) 8% polyacrylamide gel electrophoresis (PAGE) of non-specific cleavage of ssDNA in TDN-ssDNA by AsCas12a enzyme.


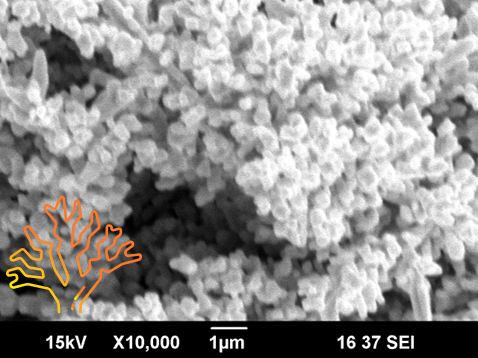


**Fig. S5.** Scanning electron microscope (SEM) image of the coralliform gold (CFAu).


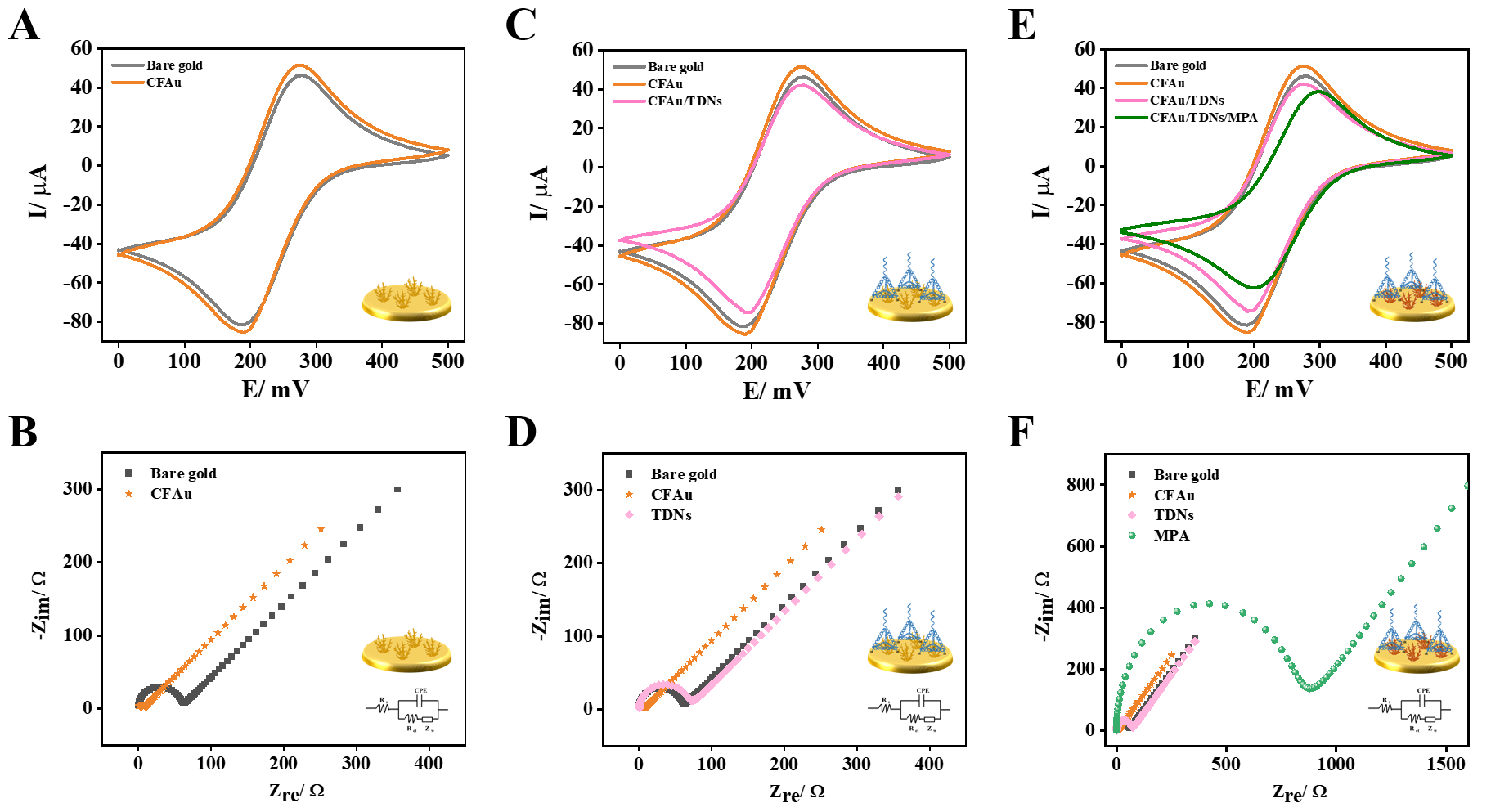


**Fig. S6.** Electrode characterization. Electrochemical characterization of stepwise modification on Au electrodes: cyclic voltammetry (CV) (A-C) and electrochemical impedance spectroscopy (EIS) (D-F) for coralliform gold (CFAu), TDNs and 3-mercaptopropionic acid (MPA) on Au electrodes in 20 mM Fe (CN)_6_^3-/4-^/PBS (10 mM, pH = 7.4). Symbols R_et_, R_S_, CPE, and Z_w_ denote electron transfer resistance, solution resistance, double-layer capacitance, and Warburg diffusion resistance, respectively.


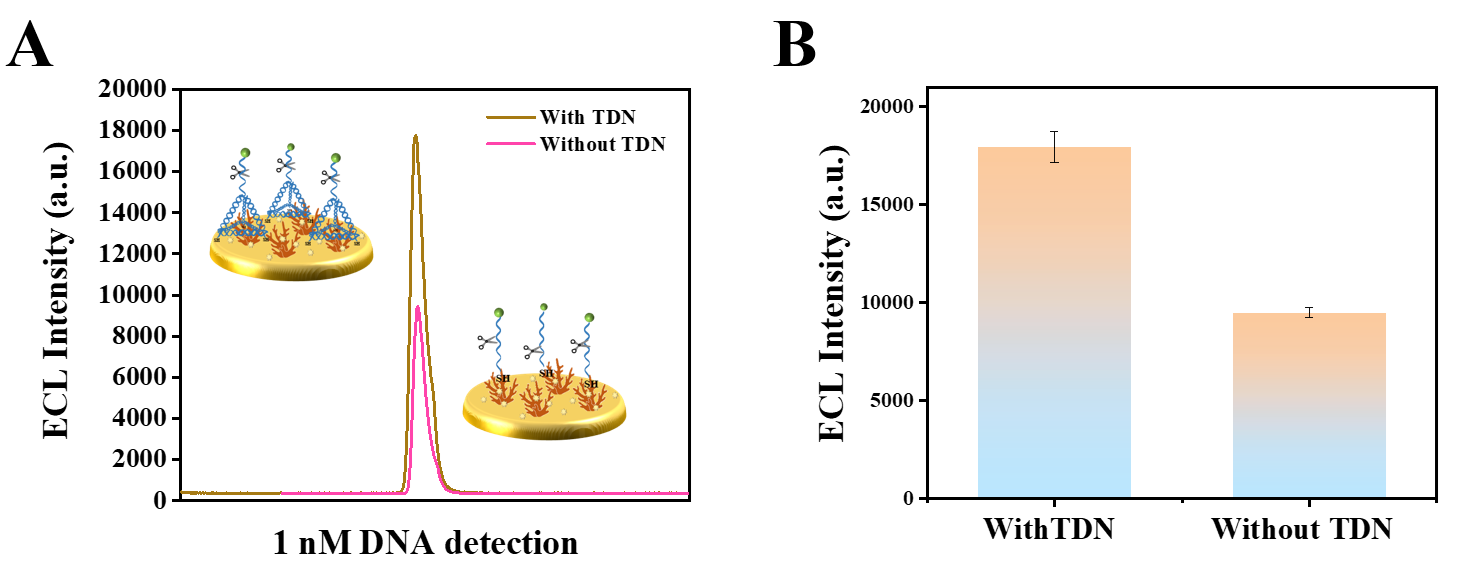


**Fig. S7.** Comparison of ECL analysis using TDN-ssDNA and ssDNA. (A) ECL analysis utilizing TDN-ssDNA and ssDNA as scaffolds for electrode connection and quencher Fc-COOH attachment, respectively. (B) Histogram of ECL analysis in (A). Error bars indicate mean ±SD, where n = 3 replicates.


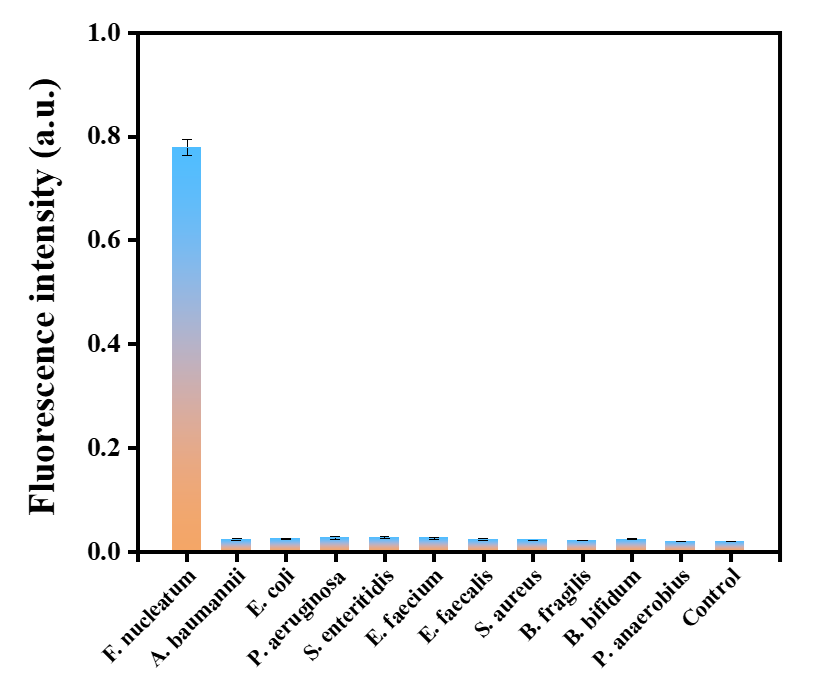


**Fig. S8.** Specificity analysis of the CRISPR/Cas12a system for *F. nucleatum* versus other bacteria by fluorescence method. Error bars indicate mean ±SD, where n = 3 replicates.

**Table S1.** Sequences for oligonucleotides and *fadA* used in this work.

| **Name** | **Sequence (5’-3’)** |
| --- | --- |
| **S1** | HS-C6-TACAGTGTCCGTACACGGCGGACTCGTAAACCAGACA  AAGCCACATGCACAACTGTAGAGAATC |
| **S2** | HS-C6-TATCACAAGACACTCATTCGTTCGCCGTGTACGGACAC  TGTACACCGTTAGCTGTCAAAAGCAT |
| **S3** | HS-C6-CTTTGTGAAAATGGAGCCAGCGATGCTTTTGACAGCTA  ACGGTTGATTCTCTACAGTTGTGCAT |
| **S4-ssDNA** | NH_2_-C6-AAAAAAAAATTAAAAAAAAACGAATGAGTGTCTTGT  GATAAGCTGGCTCCATTTTCACAAAGACGGCTTTGTCTGGTTTACGAG |
| **Target (*fadA*-TS**^a^**)** | ATGAAAAAAGTTATTTTAACATTATTTGTTTTATTATCTATTGGAATATTTGCAAATGATGAGATTATTTCAGAGTTAAAAGGACTTAATGCTGAGTATGAAAATTTAGTAAAAGAAGAAGAAGCTAGATTTCAAAAAGAAAAAGAACTTTCTGAAAGAGCAGCAGCTCAAAATGTTAAATTGGCTGAATTAAAAGCAAGCATTGAAGAAA**AATTGTTAGCAGCTCCAGAA**GAAAGAAAAACAAAATTTTTTAAAGATACTTTTGATGGTTTAGTGAAAGATTATTCAAAATATTTAAGTCAAATAAATGAAAAAATAGCTGAAAATACTGAAATAGTAAGTAATTTTGAAAAAATTCAAAAAATAAGATAG |
| **Target (*fadA*-NTS**^b^**)** | CTATCTTATTTTTTGAATTTTTTCAAAATTACTTACTATTTCAGTATTTTCAGCTATTTTTTCATTTATTTGACTTAAATATTTTGAATAATCTTTCACTAAACCATCAAAAGTATCTTTAAAAAATTTTGTTTTTCTTTC**TTCTGGAGCTGCTAACAATT**TTTCTTCAATGCTTGCTTTTAATTCAGCCAATTTAACATTTTGAGCTGCTGCTCTTTCAGAAAGTTCTTTTTCTTTTTGAAATCTAGCTTCTTCTTCTTTTACTAAATTTTCATACTCAGCATTAAGTCCTTTTAACTCTGAAATAATCTCATCATTTGCAAATATTCCAATAGATAATAAAACAAATAATGTTAAAATAACTTTTTTCAT |
| ***fadA*-PCR-F** | TGTAAAACGACGCGCCAGTGGATCC |
| ***fadA*-PCR-R** | TATGCTTCCGGCTCGTATGTTGTGTG |
| **ssDNA-FQ reporter** | FAM-TTAATT-BHQ1 |
| **ssDNA** | GGATCGCAGAGGCGCAGTCCATCAGCGATTCGAGTCTCGTGGCGAGGGGAGCCAAATCTTACTAAT |
| **crRNA** | UAAUUUCUACUCUUGUAGAU**UUCUGGAGCUGCUAACAAUU** |

a. TS: target strand; b. NTS: non-target strand.

**Table S2.** Linear equation, detection range, and sensitivity of the two detection methods.

|  | **Fluorescence detection** | **ECL detection** |
| --- | --- | --- |
| ***fadA* detection** | | |
| **Linear equation** | I _F_ = 0.61008 LgC (nM) + 0.74221 (R^2^ = 0.9840) | I_ECL_ = 3219.22 LgC (nM) + 17526.1 (R^2^ = 0.9991) |
| **Linearity range** | 0.078-2.5nM | 10^-5^-100 nM |
| **Sensitivity** | 0.61008 | 3219.22 |
| ***F. nucleatum* detection** | | |
| **Linear equation** | I _F_ = 0.32003 LgC (CFU/mL) - 1.40041 (R^2^ = 0.9956) | I_ECL_ = 1298.86 LgC (CFU/mL) - 3.77 (R^2^ = 0.986) |
| **Linearity range** | 39063-1250000 CFU/mL | 1-10^8^ CFU/mL |
| **Sensitivity** | 0.32003 | 1434.28 |

**Table S3.** A comparison of recently reported electrochemical analysis techniques based on the CRISPR/Cas system.

| **Year** | **CRISPR-Cas Enzyme** | **Target** | **Amplification Methods** | **Assay Time (min)** | **Detection Range** | **LOD**^a^ | **Ref.** |
| --- | --- | --- | --- | --- | --- | --- | --- |
| **2019** | Cas13a | miRNA-19b | No | 240 | - | 10^4^ fM | [7] |
| **2019** | Cas12a | *HPV*^b^*-16* | No | 120 | - | 5×10^4^ fM | [8] |
| **2020** | Cas12a | *HPV-16* and *-18* | CHA^c^ | 90 | 5×10^4^-10^8^ fM | 3×10^4^ fM | [9] |
| **2021** | Cas12a | *Listeria monocytogenes* | RAA^d^ | 90 | 68-6.8×10^-5^ fM  26-2.6×10^9^ CFU/mL | 6.8×10^-4^ fM  26 CFU/mL | [10] |
| **2021** | Cas12a | *Methicillin-resistant Staphylococcus aureus* | No | 90 | 10-10^5^ fM | 3.5 fM | [11] |
| **2021** | Cas12a | *HPV-16* | No | >195 | 100-10^7^ fM | 100 fM | [12] |
| **2021** | Cas12a | *Escherichia coli O157:H7* | PER^e^ | 155 | 10-10^6^ CFU/mL | 19 CFU/mL | [13] |
| **2022** | Cas13a | miRNA-19b | No | 180 | 10^4^-10^7^ fM | 10^4^ fM | [14] |
| **2022** | Cas12a | *Lachnospiraceae bacterium* | HCR^f^ | >200 | 10^4^-10^8^ CFU/mL | 20 CFU/mL | [15] |
| **2023** | Cas12a | miRNA-31 | ISPSA^g^ | 110 | ∼2200-fold-∼270000-fold | 0.31 fM-560 fM | [16] |
| **2025** | Cas12a | *fadA* and *Fusobacterium nucleatum* | No | 105 | 10-10^8^ fM  1-10^8^ CFU/mL | 10 fM  1 CFU/mL | This work |

a. LOD: limit of detection; b. HPV: human papillomavirus; c. CHA: catalytic hairpin assembly; d. RAA: recombinase-assisted amplification; e. PER: primer exchange reaction; f. HCR: hybridization chain reaction; g. ISPSA: invading stacking primer signal amplification.

# References

[1] Kim S, Cho M, Lee Y. Iridium Oxide Dendrite as a Highly Efficient Dual Electro-Catalyst for Water Splitting and Sensing of H2O2. J Electrochem Soc. 2017;164.

[2] Qin J, Kim S, Cho M, Lee Y. Hierarchical and ultra-sensitive amyloid beta oligomer sensor for practical applications. Chem Eng J. 2020;401126055. <https://doi.org/10.1016/j.cej.2020.126055>

[3] Laskin J, Wang P. Charge retention by organometallic dications on self-assembled monolayer surfaces. Int J Mass Spectrom. 2014;365-366187-193. <https://doi.org/10.1016/j.ijms.2014.01.012>

[4] Hadjar O, Futrell J H, Laskin J. First Observation of Charge Reduction and Desorption Kinetics of Multiply Protonated Peptides Soft Landed onto Self-Assembled Monolayer Surfaces. J Phys Chem C. 2007;111(49):18220-18225. 10.1021/jp075293y

[5] Alvarez J, Cooks R G, Barlow S E, Gaspar D J, Futrell J H, Laskin J. Preparation and in Situ Characterization of Surfaces Using Soft Landing in a Fourier Transform Ion Cyclotron Resonance Mass Spectrometer. Anal Chem. 2005;77(11):3452-3460. 10.1021/ac0481349

[6] Sha H, Zhang Y, Wang Y, Ke H, Xiong X, Xue H, Jia N. Electroluminescent aptasensor based on RuSiO2 nanoparticles for detection cytochrome c using ferrocene as quenching probe. Biosens Bioelectron. 2019;132203-209. <https://doi.org/10.1016/j.bios.2019.03.004>

[7] Bruch R, Baaske J, Chatelle C, Meirich M, Madlener S, Weber W, Dincer C, Urban G A. CRISPR/Cas13a-Powered Electrochemical Microfluidic Biosensor for Nucleic Acid Amplification-Free miRNA Diagnostics. Adv Mater. 2019;31(51):1905311. <https://doi.org/10.1002/adma.201905311>

[8] Dai Y, Somoza R A, Wang L, Welter J F, Li Y, Caplan A I, Liu C C. Exploring the Trans-Cleavage Activity of CRISPR-Cas12a (cpf1) for the Development of a Universal Electrochemical Biosensor. Angew Chem Int Ed. 2019;58(48):17399-17405. 10.1002/anie.201910772

[9] Zhang D, Yan Y, Que H, Yang T, Cheng X, Ding S, Zhang X, Cheng W. CRISPR/Cas12a-Mediated Interfacial Cleaving of Hairpin DNA Reporter for Electrochemical Nucleic Acid Sensing. ACS Sens. 2020;5(2):557-562. 10.1021/acssensors.9b02461

[10] Li F, Ye Q, Chen M, Zhou B, Zhang J, Pang R, Xue L, Wang J, Zeng H, Wu S, et al. An ultrasensitive CRISPR/Cas12a based electrochemical biosensor for Listeria monocytogenes detection. Biosens Bioelectron. 2021;179113073. <https://doi.org/10.1016/j.bios.2021.113073>

[11] Suea-Ngam A, Howes P D, deMello A J. An amplification-free ultra-sensitive electrochemical CRISPR/Cas biosensor for drug-resistant bacteria detection. Chem Sci. 2021;12(38):12733-12743. 10.1039/d1sc02197d

[12] Su J, Ke Y, Maboyi N, Zhi X, Yan S, Li F, Zhao B, Jia X, Song S, Ding X. CRISPR/Cas12a Powered DNA Framework-Supported Electrochemical Biosensing Platform for Ultrasensitive Nucleic Acid Analysis. Small Methods. 2021;5(12):2100935. <https://doi.org/10.1002/smtd.202100935>

[13] Bu S, Liu X, Wang Z, Wei H, Yu S, Li Z, Hao Z, Liu W, Wan J. Ultrasensitive detection of pathogenic bacteria by CRISPR/Cas12a coupling with a primer exchange reaction. Sensor Actuat B: Chem. 2021;347130630. <https://doi.org/10.1016/j.snb.2021.130630>

[14] Xu Y, Wang C, Liu G, Zhao X, Qian Q, Li S, Mi X. Tetrahedral DNA framework based CRISPR electrochemical biosensor for amplification-free miRNA detection. Biosens Bioelectron. 2022;217114671. <https://doi.org/10.1016/j.bios.2022.114671>

[15] Liu X, Bu S, Feng J, Wei H, Wang Z, Li X, Zhou H, He X, Wan J. Electrochemical biosensor for detecting pathogenic bacteria based on a hybridization chain reaction and CRISPR-Cas12a. Anal Bioanal Chem. 2022;414(2):1073-1080. 10.1007/s00216-021-03733-6

[16] Wang H, Li Y L, Fan Y J, Dong J X, Ren X, Ma H, Wu D, Gao Z F, Wei Q, Xia F. DNA Tile and Invading Stacking Primer-Assisted CRISPR–Cas12a Multiple Amplification System for Entropy-Driven Electrochemical Detection of MicroRNA with Tunable Sensitivity. Anal Chem. 2023;95(36):13659-13667. 10.1021/acs.analchem.3c02603
